# SETD1A regulates transcriptional pause release of heme biosynthesis genes in leukemia

**DOI:** 10.1016/j.celrep.2022.111727

**Published:** 2022-11-29

**Authors:** Takayuki Hoshii, Sarah Perlee, Sota Kikuchi, Bahityar Rahmutulla, Masaki Fukuyo, Takeshi Masuda, Sumio Ohtsuki, Tomoyoshi Soga, Behnam Nabet, Atsushi Kaneda

**Affiliations:** 1 Department of Molecular Oncology, Graduate School of Medicine, Chiba University, Chiba 260-8670, Japan; 2 Department of Cancer Biology and Genetics, Memorial Sloan Kettering Cancer Center, New York, NY 10065, USA; 3 Gerstner Graduate School of Biomedical Sciences, Memorial Sloan Kettering Cancer Center, New York, NY 10065, USA; 4 Laboratory of Pharmaceutical Microbiology, Faculty of Life Sciences, Kumamoto University, Kumamoto 862-0973, Japan; 5 Institute for Advanced Biosciences, Keio University, Yamagata 997-0035, Japan; 6 Human Biology Division, Fred Hutchinson Cancer Center, Seattle, WA 98109, USA; 7 Lead contact

## Abstract

Histone methyltransferase SETD1A is critical for acute myeloid leukemia (AML) cell survival, but the molecular mechanism driving SETD1A gene regulation remains elusive. To delineate the role of SETD1A, we utilize a protein degrader technology to induce rapid SETD1A degradation in AML cell lines. SETD1A degradation results in immediate downregulation of transcripts associated with DNA repair and heme biosynthesis pathways. CRISPR-based functional analyses and metabolomics reveal an essential role of SETD1A to maintain mitochondrial respiration in AML cells. These SETD1A targets are enriched in head-to-head (H2H) genes. SETD1A degradation disrupts a non-enzymatic SETD1A domain-dependent cyclin K function, increases the Ser5P RNA polymerase II (RNAPII) at the transcriptional start site (TSS), and induces the promoter-proximal pausing of RNAPII in a strand-specific manner. This study reveals a non-enzymatic role for SETD1A in transcriptional pause release and provides insight into the mechanism of RNAPII pausing and its function in cancer.

## In brief

Hoshii et al. report the role of histone-modifying enzyme SETD1A in the regulation of mitochondrial respiration in acute myeloid leukemia cells. They further show a non-enzymatic function of SETD1A is required for transcriptional pause release of heme biosynthesis genes.

## Graphical Abstract

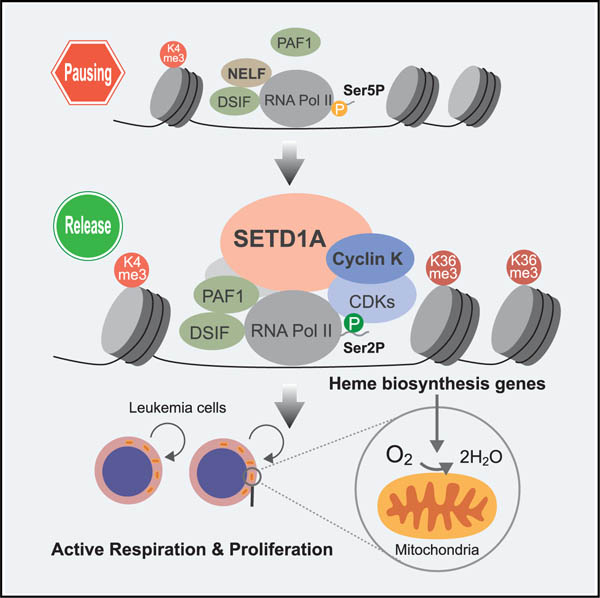

## INTRODUCTION

Histone H3 lysine 4 tri-methylation (H3K4me3) is observed around the transcriptional start sites (TSSs) of active genes and the gene body with enhanced transcriptional consistency.^3^ The mixed-lineage leukemia (MLL) family of histone methyltransferases, including SETD1A, are key regulators of H3K4 methylation. Many studies have linked the functional role of SETD1A to H3K4me3 level in embryonic stem cells (ESCs), tumor cells, and neural progenitor cells.^4–9^ In contrast, our group and others previously identified a non-enzymatic role of SETD1A in leukemia, sarcoma, and ESCs.^7,10^ The functional location on SETD1A (FLOS) domain functions as a non-enzymatic domain of SETD1A crucial for leukemia cell survival.^7^ Binding between FLOS and cyclin K (encoded by *CCNK*) facilitates the active transcription of DNA-repair-associated genes, but the molecular mechanism of how this interaction regulates specific gene sets remains unclear.

Cyclin K is a co-factor of cyclin-dependent kinases (CDKs) and has been shown to interact with CDK9/12/13.^11–13^ CDK9 is a catalytic subunit of positive elongation factor b (P-TEFb) that phosphorylates the Ser2/5 of the RNA polymerase II (RNAPII) C-terminal domain (CTD) at the TSS. P-TEFb is also required to overcome the effects of negative elongation factors to enable productive elongation.^14,15^ In contrast, CDK12/13 are known to promote transcription elongation by phosphorylating Ser2 on RNAPII at the gene body. CDK12/13 are functionally redundant *in vitro*, but *in vivo*, knockdown of CDK12 perturbs genes of the DNA repair pathway, while loss of CDK13 results in changes to cellular metabolism.^16,17^ Since the cyclin K-CDK12 axis is important for regulation of DNA repair genes, SETD1A-cyclin K complex may function as a potential regulator of CDK12.^7^ In contrast, SETD1A and CDK12 display different genome-wide localization profiles, with SETD1A found at TSSs and CDK12 at gene bodies, suggesting that these factors may modulate disparate transcriptional steps. Because of the limited information on the SETD1A-cyclin K complex, further studies on the complex are required to elucidate the role of SETD1A in CDK regulation.

CDKs are promising targets for cancer therapy. The development of selective small-molecule inhibitors against CDKs demonstrated the potential success of CDK inhibitors in clinical practice.^18,19^ CDK12/13 inhibitors are still in the preclinical stage, but these compounds effectively block tumor growth in patient-derived xenografts.^20,21^ Histone modification enzymes are also attractive drug targets, but there are no specific compounds for SETD1A. One innovative tool for drug discovery that enables selective targeting against non-enzymatic protein/domains, such as SETD1A FLOS, is proteolysis-targeting chimera (PROTAC).^22,23^ By utilizing this technology, we can block the molecular functions of undruggable targets and evaluate the kinetics of the molecular cascade following inhibition.

In this study, we first established the PROTAC model of SETD1A in leukemia cell lines. We report a non-catalytic role for SETD1A in releasing paused RNAPII to regulate genes of the heme biosynthesis pathway. We show that SETD1A degradation immediately downregulates heme biosynthesis pathway genes, which are required for functional cellular metabolism and leukemia cell proliferation. Taken together, our study reveals that SETD1A plays a non-enzymatic role in the regulation of RNAPII activity for productive elongation at TSS and suggests a potential therapeutic window for cancers with transcriptional addiction.

## RESULTS

### dTAG-based SETD1A protein degradation induces apoptosis in leukemia

**Figure 1.**
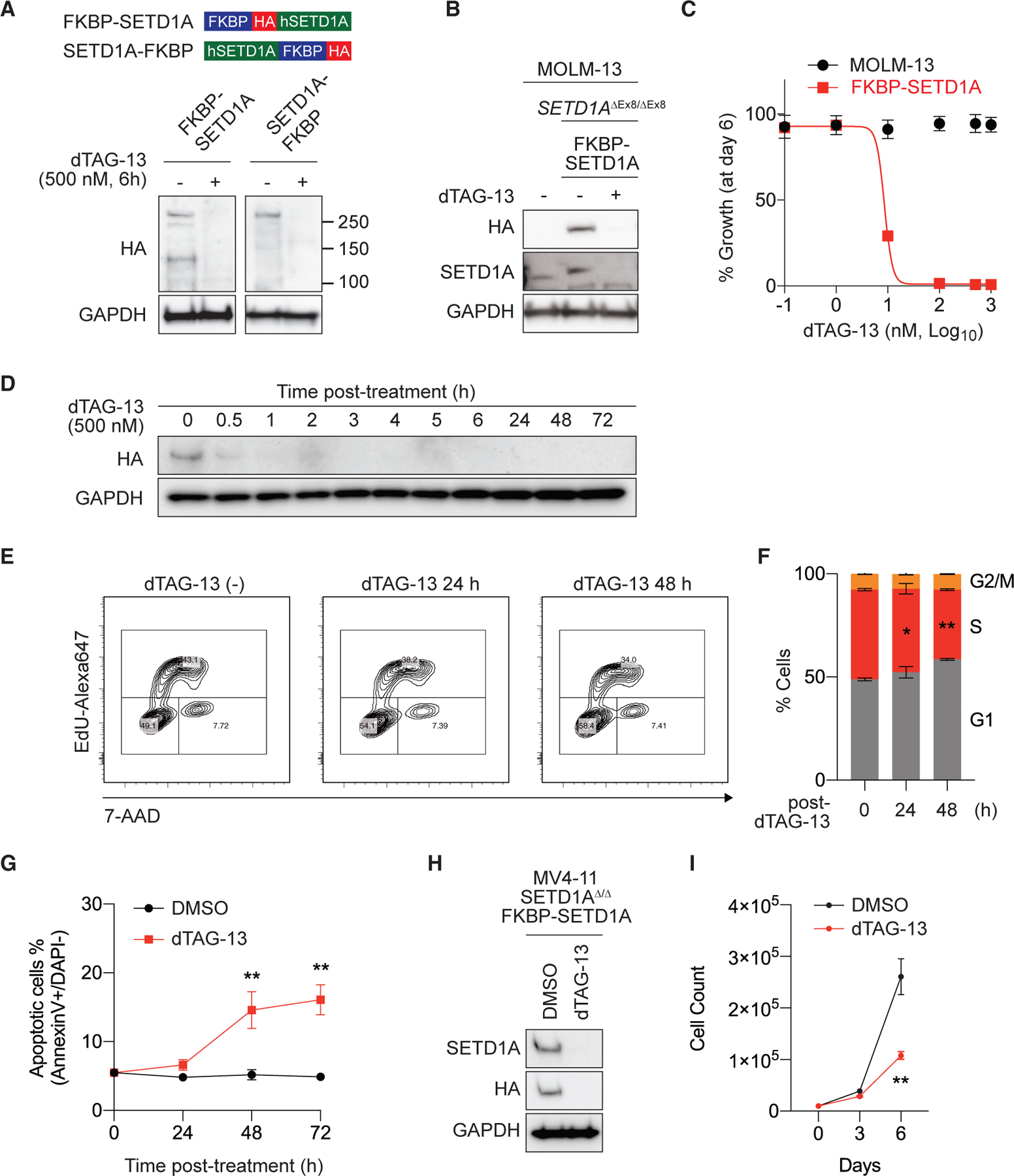
FKBP^F36V^-tagged SETD1A degradation by dTAG-13 induces apoptosis in leukemia cell lines (A) FKBP12^F36V^-HA-SETD1A (FKBP-SETD1A) or SETD1A-FKBP12^F36V^-HA (SETD1A-FKBP) fusions were expressed in *Setd1a*^Δ/Δ^;CreER MLL-AF9 leukemia cells and treated with 500 nM dTAG-13 for 6 h. FKBP-tagged SETD1A protein levels were quantified with an anti-HA antibody. (B) FKBP-SETD1A was expressed in MOLM-13 cell line, and endogenous *SETD1A* was deleted by CRISPR-Cas9. Cells were treated with dTAG-13 for 6 h, and protein levels were quantified. (C) MOLM-13 cells and FKBP-SETD1A-expressing/endogenous *SETD1A*-deleted MOLM-13 (FKBP-SETD1A) cells were treated with different doses of dTAG-13 for 6 days. (D) FKBP-SETD1A cells were treated with dTAG-13 for indicated times, and proteins were quantified by western blot analysis. (E and F) Cell cycling of FKBP-SETD1A cells was measured at 24 or 48 h post-dTAG-13 treatment. EdU-incorporated cell frequency was measured by flow cytometer (E). Ratio of each cell-cycle stage is shown (F). (G) Annexin V+ DAPI− population in DMSO- or dTAG-13-treated FKBP-SETD1A cells was analyzed at 24 h intervals after treatment. Representative data from 3 independent experiments with 3 biological replicates are shown. (H) FKBP-SETD1A was expressed in the MV4–11 cell line, and endogenous SETD1A was deleted by CRISPR-Cas9. Cells were treated with dTAG-13 for 24 h, and protein levels were quantified. (I) FKBP-SETD1A-expressing MV4–11 cells were cultured and counted every 3 days following dTAG-13 treatment. Representative data from 2 independent experiments with 3 biological replicates are shown. Data are represented as mean ± SD. See also Figure S1.

To evaluate the primary response of leukemia cells to SETD1A depletion, we constructed FKBP12^F36V^-HA-tagged SETD1A expression vectors (Figure 1A). First, we transduced these vectors into mouse inducible *Setd1a* knockout MLL-r leukemia cells and established *Setd1a* knockout cells, which can grow with the replaced exogenous FKBP12^F36V^-HA-tagged SETD1A. We validated the effect of PROTAC compound dTAG-13 treatment by western blot analysis and cell growth assay (Figures 1A and S1A). We observed comparable degradation between N terminus and C terminus tagged SETD1A but selected the N terminus FKBP12^F36V^-HA-tagged SETD1A (FKBP-SETD1A) for subsequent experiments due to a more robust response to dTAG-13 as observed by cell growth assay. Next, we established MOLM-13 human MLL-r leukemia cells harboring both Cas9 and exogenous FKBP-SETD1A. We disrupted the endogenous SETD1A in these cells using a single guide RNA (sgRNA) that targets the intron-exon junction of endogenous *SETD1A* (Figures 1B and S1B–S1D). The IC50 value of dTAG-13 on the FKBP-SETD1A cells was 8.6 nM, with complete suppression of cell growth at 6 days post-treatment occurring with a dTAG-13 concentration of 100 nM (Figure 1C). dTAG-13 (500 nM) induced remarkable SETD1A degradation after only 30 min (Figure 1D). Treatment of FKBP-SETD1A cells with dTAG-13 minimally suppressed the cell cycling at 24 h post-treatment and led to significant apoptosis by 48 h in the MOLM-13 cell line (Figures 1E–1G). To validate this system in another MLL-r leukemia cell line, we generated a MV4–11 leukemia cell line expressing FKBP-SETD1A (Figure 1H). This cell line showed growth retardation at 6 days post-dTAG-13 treatment (Figure 1I). These data indicate that in human acute myeloid leukemia (AML) cells, 24 h post-dTAG-13 treatment is the ideal time point for gene expression analysis as we observe complete degradation of FKBP-SETD1A prior to significant cell-cycle arrest and apoptosis.

### SETD1A directly regulates the heme biosynthesis pathway

**Figure 2.**
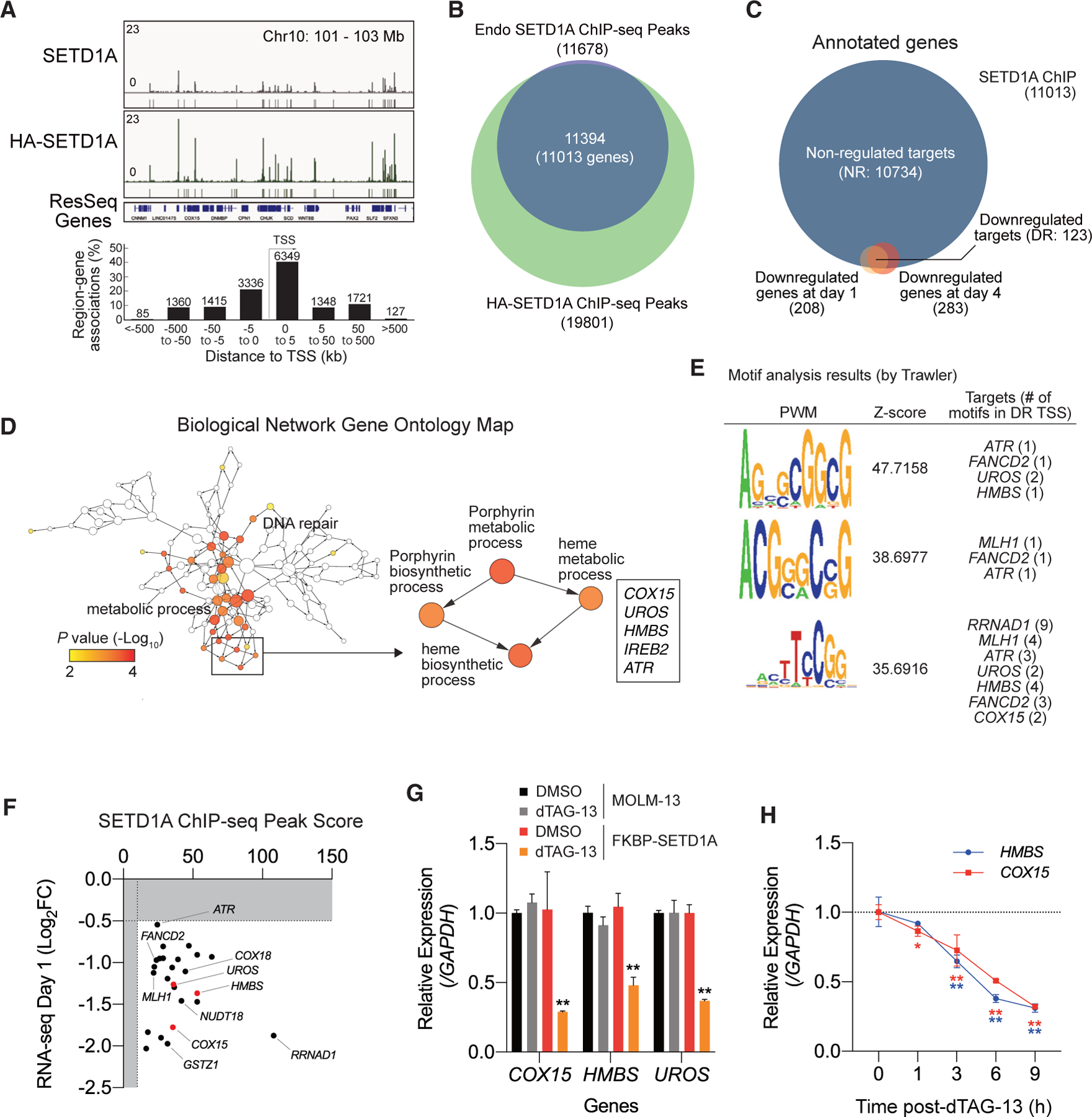
SETD1A degradation immediately decreases the expression of heme biosynthesis genes (A) Chromatin distribution of endogenous SETD1A or exogenous HA-tagged FKBP-SETD1A was analyzed from MOLM-13 cells or FKBP-SETD1A cells, respectively. Genome browser view at human chromosome 10 (chr10) is shown (top). The distribution of overlapped peaks around the TSS was analyzed with GREAT tool (bottom). (B) The overlap between endogenous SETD1A ChIP-seq peaks and exogenous FKBP-SETD1A ChIP-seq peaks is shown. The overlapped region is labeled as SETD1A ChIP peaks, and 11,013 genes were annotated by GREAT tool. (C) The overlap among SETD1A ChIP-annotated genes and the downregulated genes (log_2_ fold change < −0.5, adjusted p value < 0.05) at 1 and 4 days post-dTAG-13 treatment in FKBP-SETD1A cells was analyzed. 123 commonly downregulated (DR) targets and 10,734 non-regulated (NR) targets of SETD1A were identified. (D) GO analysis of DR genes was performed, and the biological network Gene Ontology map was created by Cytoscape. Heme biosynthetic process is high-lighted, and associated genes are shown in the box. (E) Motifs within the SETD1A peaks in DR genes were analyzed by Trawler. Position weight matrices (PWM), *Z* scores of discovered motifs, and the number of motifs in representative DR TSSs are shown. (F) Association of SETD1A peak score and log_2_ fold change (log_2_FC) from RNA-seq at 1 day post-dTAG-13 treatment is shown. Heme biosynthesis genes (*COX15*, *HMBS*, and *UROS*) are shown in red dots. (G) MOLM-13 cells and FKBP-SETD1A cells were treated with DMSO or dTAG-13 for 24 h, and RNA levels of heme biosynthesis genes were quantified by qRT-PCR. (H) Time course semi-quantification analysis for *HMBS* and *COX15* expression after SETD1A degradation. RNA was extracted at the indicated time points from FKBP-SETD1A cells treated with dTAG-13. Two independent experiments with 3 biological replicates are performed. Data are represented as mean ± SD. See also Figure S2.

Using the HA-tagged FKBP-SETD1A protein, we were able to detect chromatin binding of the exogenous SETD1A in the transduced leukemia cells. We observed a strong overlap between endogenous and exogenous SETD1A peaks and saw higher signal intensity of HA-tagged SETD1A compared with endogenous SETD1A (Figures 2A and 2B). These overlapped SETD1A peaks are observed around TSSs, with the peak centers most abundant at ±5 kb, especially at 0 to 5 kb, from TSSs (Figure 2A). We also performed RNA sequencing (RNA-seq) analyses at 1 or 4 days post-dTAG-13 treatment and identified genes that are downregulated after dTAG-13 treatment (Figures 2C and S2A). We compared our list of annotated genes that contained SETD1A peaks with those that were downregulated following dTAG-13 treatment and identified a list of 123 overlapping genes as the potential downstream targets of SETD1A in human leukemia cells (Figure 2C). In this study, we labeled the 123 downregulated targets and 10,734 non-regulated targets of SETD1A as DR and NR, respectively. The SETD1A peaks that are annotated to DR genes were remarkably enriched around the TSS (Figure S2B). To understand the functions of these bona fide SETD1A targets, we analyzed the gene list with Gene Ontology (GO) analyses and identified the enrichment of genes associated with DNA repair and heme biosynthesis processes (Figures 2D and S2C). We also confirmed the downregulation of genes in heme biosynthesis processes (porphyrin metabolism) in SETD1A-deficient MV4–11 cells (Figures S2D and S2E). From motif analysis of the TSS regions of DR genes, we identified that SETD1A binds to CG-rich sequences (Figure 2E). Chromatin immunoprecipitation (ChIP)-seq peak scores from the endogenous SETD1A showed that heme biosynthesis genes (*COX15*, *HMBS*, and *UROS*) have stronger SETD1A binding than DNA-repair-pathway-associated genes (*FANCD2*, *MLH1*, and *ATR*), which are the known SETD1A targets (Figure 2F). The downregulation of *COX15* expression by SETD1A disruption is also observed in non-MLL-r leukemia cell lines (U937 and K562) as well as sarcoma cell lines (A673 and RH30) (Figures S2F and S2G). Off-target effects of dTAG-13 did not influence the expression of heme biosynthesis genes in the parental MOLM-13 leukemia cells (Figure 2G). In human AML cell lines, expression of *SETD1A* and *COX15*/*COX18* are highly correlated in DepMap database (DepMap, 2022; https://depmap.org/) (Figure S2H). In TCGA database, the expression of *SETD1A* is positively correlated with the expression of *HMBS*/*UROS* but is negatively correlated with *COX15*/*COX18* (Figures S2I and S2J). These results imply that leukemic heterogeneity or the secondary compensatory upregulation of heme biosynthesis genes creates a complex situation of gene-expression profiles *in vivo*. We evaluated the gene-expression kinetics of *HMBS* and *COX15* at 1, 3, 6, and 9 h post-dTAG-13 treatment (Figure 2H). Expression of both genes was immediately downregulated at 1 h and significantly downregulated at 3 h post-dTAG-13 treatment. These data revealed that the heme biosynthesis pathway is regulated by SETD1A-dependent transcriptional activation in AML cells.

### Heme A biosynthesis pathway genes are essential for leukemia cell proliferation

**Figure 3.**
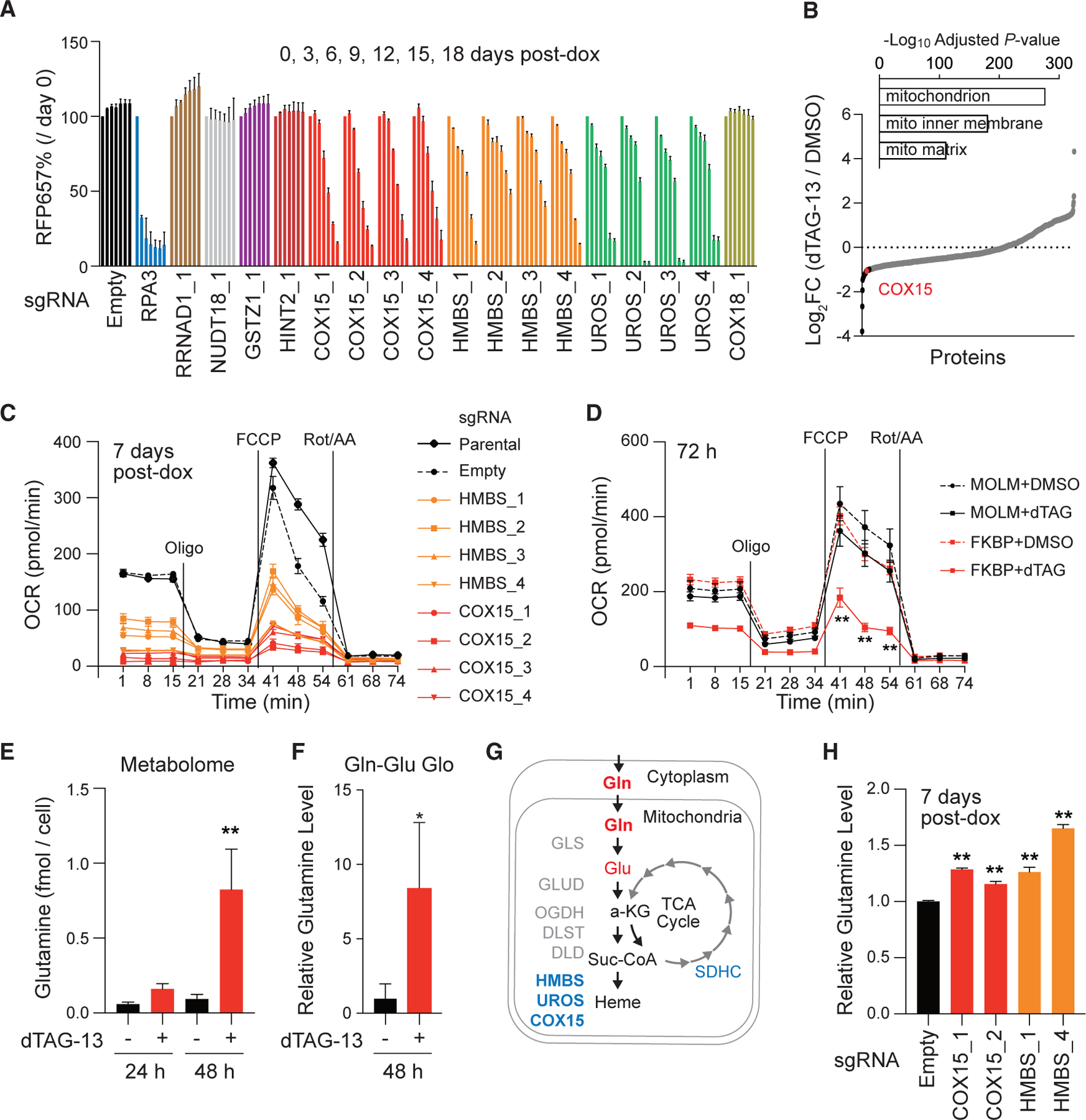
Genes of the heme biosynthesis pathway are essential for the mitochondrial respiration and glutamine metabolism in leukemia cells (A) sgRNAs for SETD1A target candidates were transfected into iCas9-expressing MOLM-13 leukemia cells. Four independent sgRNAs against each candidate were tested. For each sgRNA, data are shown from 0 to 18 days, left to right. Representative data from 2 independent experiments with 3 biological replicates are shown. (B) Mitochondrial proteomics were performed with purified mitochondria from dTAG-13-treated FKBP-SETD1A cells at 24 h. GO term analysis with identified proteins were shown on the top. Reduced proteins (log_2_FC < −1) are shown in black dots. COX15 is shown as a red dot. (C) OCR in *COX15*- or *HMBS*-knockout cells were evaluated by flux analyzer at 7 days post-doxycycline (dox) treatment. Two independent experiments with 5 biological replicates are performed. (D) OCR in MOLM-13 and FKBP-SETD1A cells were evaluated by flux analyzer at 72 h post-dTAG-13 treatment. Two independent experiments with 5 biological replicates are performed. (E) Metabolomics was performed at 24 and 48 h post-dTAG-13 treatment in FKBP-SETD1A cells. Concentrations of glutamine per cell are shown. Samples from 3 independent experiments are analyzed. (F) Intracellular glutamine levels were measured by glutamine (Gln)-Glu Glo assay at 48 h post-dTAG-13 treatment in FKBP-SETD1A cells. Representative data from 2 independent experiments with 3 biological replicates are shown. (G) Schematic of Gln metabolism and heme biosynthesis pathway are shown. (H) Intracellular Gln levels were measured by Gln-Glu Glo assay at 7 days post-dox treatment in *COX15*- or *HMBS*-knockout cells. Relative Gln levels against empty sgRNA-expressing control are shown. Data are represented as mean ± SD. See also Figure S3.

To validate the function of SETD1A target genes (*COX15*, *HMBS*, *UROS*, *RRNAD1*, *NUDT18*, *GSTZ1*, *HINT2*, and *COX18*) in leukemia cells, we performed a CRISPR-based competitive cell proliferation assay in doxycycline-inducible Cas9-expressing MOLM-13 leukemia cells (Figures 3A and S3A). We found a strong dependency for COX15, HMBS, and UROS in leukemia cells (Figure 3A). These three genes are all required for heme A biosynthesis, which is an essential metabolite for reduction of oxygen on the inner mitochondrial membrane by complex IV. To monitor mitochondrial phenotypes, we performed mass spectrometry analysis against purified mitochondria and detected the reduction of COX15 protein in mitochondria of dTAG-13-treated cells (Figures 3B, S3B, and S3C). Mitochondrial proteomics also indicated that the oxidative phosphorylation (OXPHOS) pathway is disrupted in SETD1A-deficient cells (Figure S3D). Unlike AML cells, we found that non-AML cells do not exhibit a strong dependency on COX15, suggesting that the heme biosynthesis pathway may be a specific dependency of AML cell lines (Figure S3E). To evaluate the effect of suppression of these SETD1A targets on mitochondrial oxygen consumption in leukemia cells, we measured the mitochondrial oxygen consumption rate (OCR) of sgRNA-expressing leukemia cells by flux analyzer (Figure 3C). Both *COX15* and *HMBS* knockout significantly reduced the OCR at the basal level (Figure 3C). In contrast, COX18, the other SETD1A-dependent complex IV assembly factor, was dispensable for leukemia cell growth as well as OCR (Figures 3A, S3A, and S3F). Consistent with these results, a reduction in OCR was observed in the dTAG-13-treated FKBP-SETD1A-expressing cells (Figure 3D). Reduced oxygen consumption can be compensated by upregulation of glycolysis, but SETD1A degradation did not result in a significant difference in the extracellular acidification rate, even in stress conditions (Figure S3G). ATP production was moderately reduced in SETD1A-deficient cells (Figure S3H). We labeled mitochondria with MITO-tag (OMP25-GFP) to monitor mitochondrial mass and morphology but did not observe any apparent changes to the morphology or the total mass of the mitochondria upon SETD1A degradation (Figures S3I and S3J). These results strongly suggest that SETD1A is specifically required for the maintenance of mitochondrial respiration through modulation of the heme biosynthesis pathway in AML cells.

### SETD1A is required for the glutamine metabolism in leukemia cell

To investigate the roles of SETD1A-dependent metabolic regulation in leukemia cell proliferation, we performed metabolome analysis at 24 and 48 h post-dTAG-13 treatment in FKBP-SETD1A-expressing cells. Most metabolites were unaffected by SETD1A loss, but surprisingly, glutamine (Gln) and glutamate (Glu) levels were significantly increased in SETD1A-deficient cells (Figures 3E and S3K). An increase in Gln/Glu accumulation was also observed with the cell-based Gln/Glu detection assay in SETD1A-deficient cells (Figures 3F and S3L). Gln and Glu are known ingredients for the heme biosynthesis pathway, and defective Gln metabolism was also observed in heme degradation enzyme *BLVRB*-knockout cells.^24^ We therefore hypothesized that SETD1A-deficient cells may have lower consumption of these metabolites (Figure 3G). To examine the role of the heme biosynthesis pathway in Gln accumulation, we evaluated Gln levels in *COX15*- or *HMBS*-knockout cells. As expected, the knockout of *COX15* or *HMBS* also increased the Gln level (Figure 3H). These results indicate that SETD1A promotes Gln/Glu consumption by regulating the heme biosynthesis pathway in AML cells.

### SETD1A loss increases the CTD-Ser5 phosphorylated RNAPII

**Figure 4.**
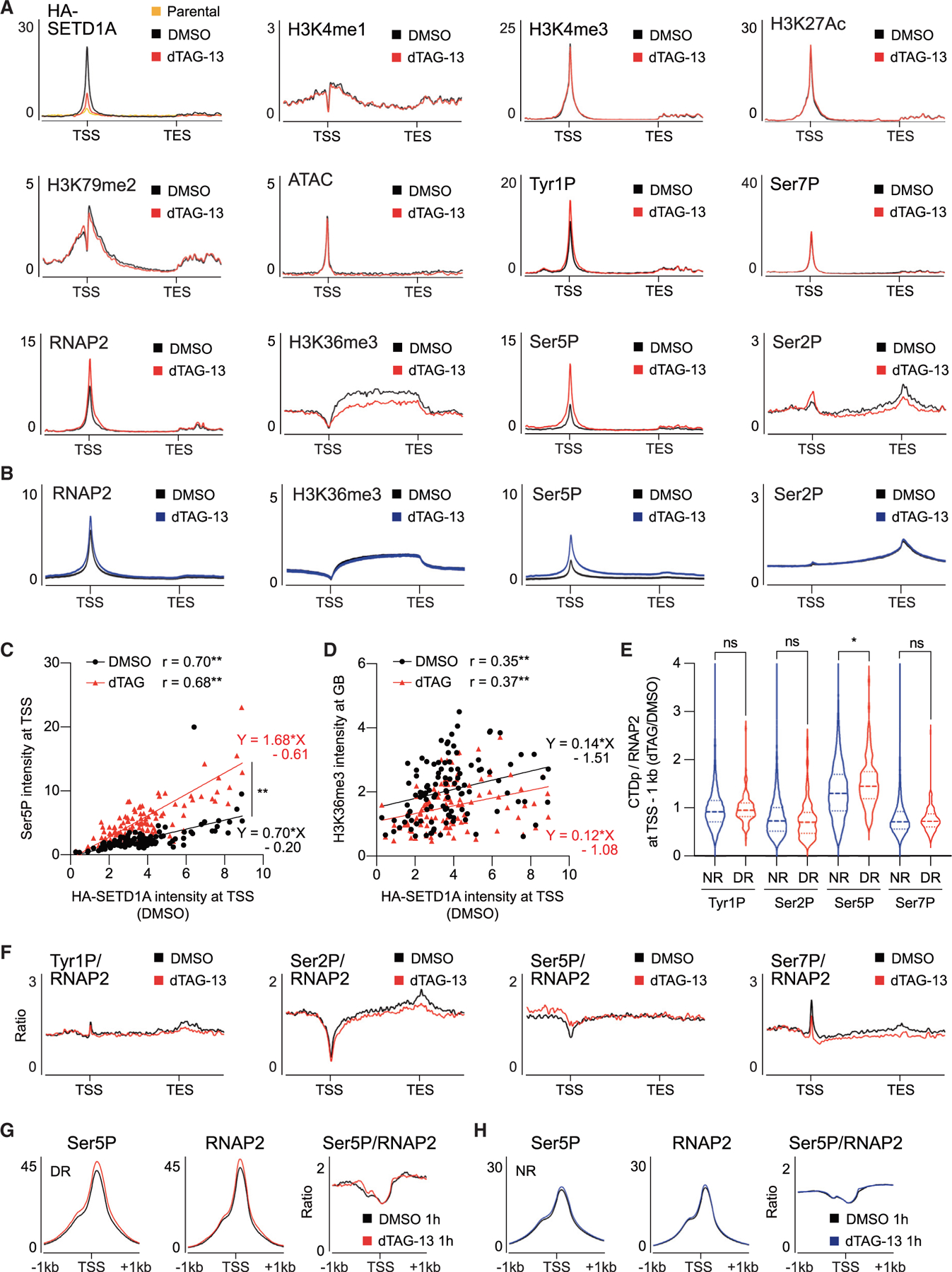
SETD1A degradation induces hyper-phosphorylation of RNAPII Ser5 (A) Average ChIP-seq signals of HA-SETD1A, H3K4me1/3, H3K27Ac, H3K79me2, ATAC, Tyr1P, Ser7P, RNAPII, H3K36me3, Ser5P, and Ser2P along DR genes and flanking regions from DMSO-(black) or dTAG-13-treated (red) cells are shown. (B) Average ChIP-seq signals of RNAPII, H3K36me3, Ser5P, and Ser2P along NR genes and flanking regions from DMSO-(black) or dTAG-13-treated (blue) cells are shown. (C and D) Correlation of ChIP-seq signal intensities between HA-SETD1A at the TSS in DMSO-treated cells and Ser5P at the TSS (C) or H3K36me3 at the gene body (D) in DMSO- or dTAG-13-treated cells are shown. Correlation coefficient (r) and p values of Spearman correlation in DMSO- or dTAG-13-treated cells are shown. The simple linear regression equations and the difference between two groups were also calculated. (E) Log_2_FC of normalized ChIP-seq signals of each RNAPII phosphorylation (Tyr1P, Ser2P, Ser5P, and Ser7P) by total RNAPII at the TSS −1 kb of NR or DR genes in DMSO- or dTAG-13-treated samples are shown. (F) ChIP-seq signals of each RNAPII phosphorylation normalized to total RNAPII along DR genes and flanking regions from DMSO-(black) or dTAG-13-treated (red) cells are shown. (G and H) Average ChIP-seq signals of Ser5P, total RNAPII, and the ratio of Ser5P/RNAPII at TSS ±1 kb of DR (H) or NR (I) genes in 1 h dTAG-13-treated cells are shown. See also Figure S4.

Our study indicates a role for SETD1A in regulation of mitochondrial respiration and metabolism, but the mechanism of how SETD1A regulates these specific gene sets in AML cells remains unclear. To investigate histone modification modulation and changes in RNAPII activity on SETD1A target genes after SETD1A depletion, we detected HA-tagged SETD1A, active histone marks (H3K4me1, H3K4me3, H3K27Ac, H3K36me3, and H3K79me2), open chromatin (assay for transposase-accessible chromatin [ATAC]), and phosphorylation states of RNAPII (total, Tyr1 phosphorylation [Tyr1P], Ser7P, Ser5P, and Ser2P) at 24 h post-dTAG-13 treatment (Figures 4A, 4B, and S4A). As we observed in *Setd1a*-knockdown NIH3T3 cells, the total RNAPII signal was significantly increased at the TSS in the dTAG-13-treated cells (Figures 4A and 4B).^7^ In contrast, histone modifications, including H3K4me1, H3K4me3, H3K27Ac, and H3K79me2, showed comparable levels across the two conditions at SETD1A target genes (Figure 4A). Consistent with the mRNA expression levels of DR genes, H3K36me3 was reduced on DR, but not NR, genes (Figures 4A and 4B). We also assessed RNAPII phosphorylation and detected a significant increase in Ser5P at TSSs on both DR and NR loci (Figures 4A and 4B). Similar to the total RNAPII signal, dTAG-13-treated cells also displayed an increase in Tyr1P at the TSS (Figure 4A). Although Ser7P is also primarily observed at the TSS, we did not detect changes in Ser7P levels at DR loci (Figure 4A). We found that the Ser2P signal, which is usually more abundant at transcript end sites (TESs) than at TSS, was decreased at the TES on DR loci in dTAG-13-treated cells (Figures 4A and 4B). ATAC-seq results revealed that the open chromatin around the TSS was not changed by SETD1A depletion (Figure 4A). Both H3K36me3 and Ser2P are well-known markers of transcriptional elongation, and we observed coordinated reduction of these signals on DR genes, suggesting that transcriptional elongation may be specifically perturbed by SETD1A loss (Figures S4A and S4B). Since the H3K36me3 level was reduced as early as 1 h post-dTAG-13 treatment, this suggests that SETD1A directly regulates transcriptional elongation at these loci (Figure S4C).

Ser5P signal intensity is well correlated with the intensity of SETD1A at the TSS, and Ser5P signals are selectively enhanced at SETD1A-binding sites upon depletion of SETD1A, indicating a role for SETD1A in the maintenance of Ser5P RNAPII level at TSSs (Figures 4C and S4D). H3K36me3 signals in the gene body are also correlated with the intensity of SETD1A at the TSS, but the slopes of simple linear regression were not significantly different after SETD1A depletion (Figures 4D and S4E). The accumulation of Ser5P signals at all SETD1A-binding sites and the locus-specific downregulation of H3K36me3 signals indicated that additional factors may contribute to the locus-specific transcriptional elongation defects in SETD1A-deficient cells. We also compared the CTD Ser phosphorylation level per total RNAPII between DR and NR genes. Ser5P showed a higher ratio at the promoter for both DR and NR genes, and DR genes showed an even higher ratio of Ser5P to NR genes in SETD1A-deficient cells. (Figures 4E, 4F, and S4F). In contrast, DR genes showed a lower ratio of Ser2P at the gene body in SETD1A-deficient cells (Figures 4F and S4F). Despite the upregulation of the Ser5P ratio at the promoter, the ratio of all phosphorylation marks was downregulated at the TES of DR genes in SETD1A-deficient cells (Figure S4G). To examine the immediate effects of SETD1A degradation on RNAPII, we analyzed the RNAPII levels starting from 1 h post-dTAG-13 treatment. Ser5P was continuously increased at the COX15 TSS from 1 to 6 h post-dTAG-13 treatment (Figure S4H). Both the amounts of Ser5P and RNAPII were increased at the TSS of DR genes at 1 h post-dTAG-13 treatment (Figures 4G and 4H). The ratio of Ser5P was increased, especially in the region surrounding the TSS of DR genes, at 1 h post-dTAG-13 treatment (Figures 4G and 4H). These data demonstrate that Ser5P RNAPII accumulation at the TSS is a hallmark of transcriptional perturbation of SETD1A target genes.

### SETD1A is required for the release of paused RNAPII on head-to-head (H2H) genes

**Figure 5.**
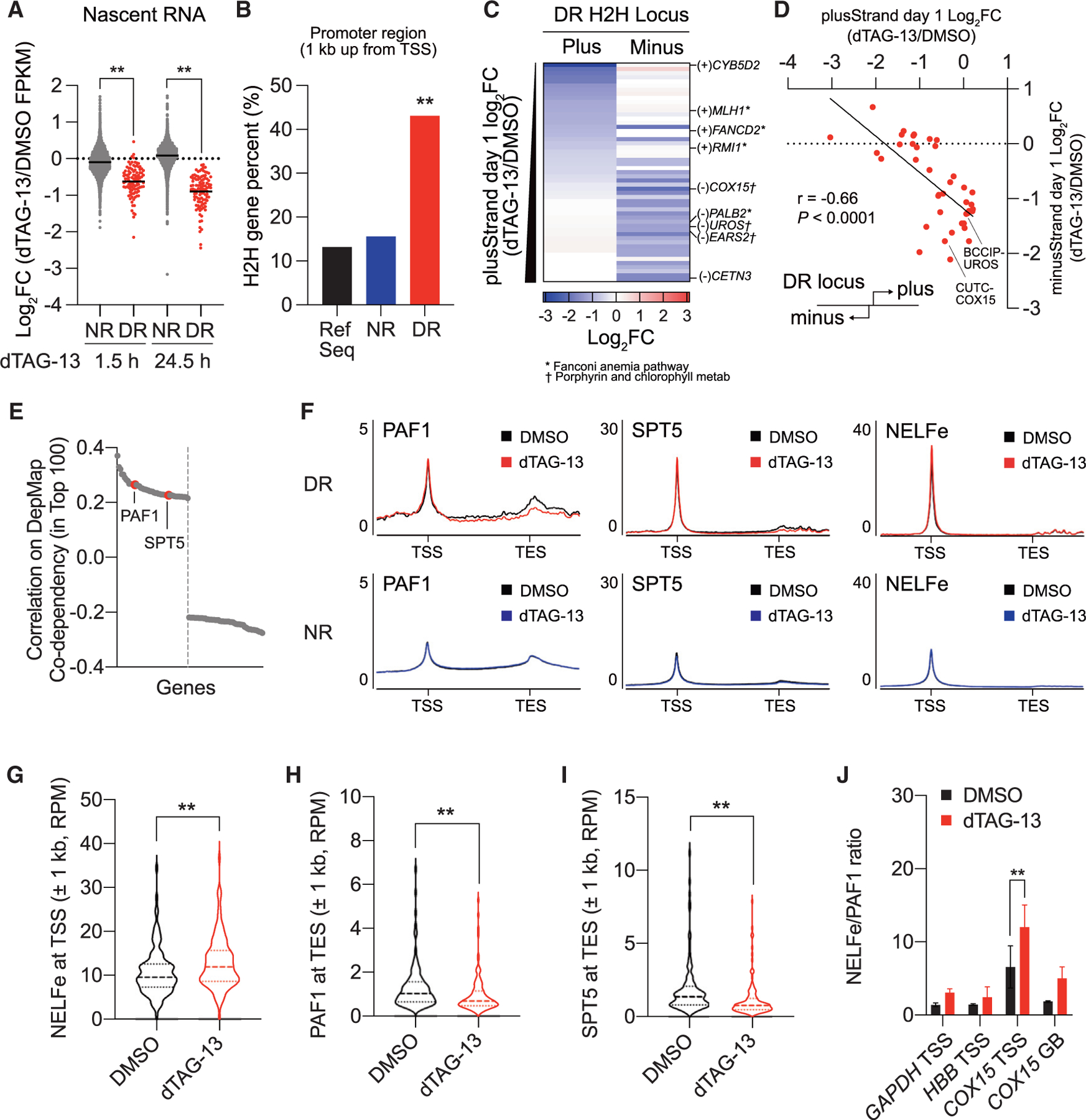
SETD1A degradation causes a pause release defect in H2H genes (A) Nascent RNA was enriched by EU labeling and quantified by RNA-seq. One and 24 h DMSO- or dTAG-13-treated cells were additionally labeled with EU for 0.5 h (total 1.5 and 24.5 h dTAG-13 treatment). Violin plot for the log_2_FC between DMSO- and dTAG-13-treated groups in NR and DR genes is shown. (B) H2H loci in Refseq, NR or DR genes are identified and their percentages in each gene list are shown. We identified 1,921 H2H loci from RefSeq by measuring the overlap of the 1 kb upstream region from the TSS. 53 and 1,421 H2H loci were annotated from 123 DR genes and 11,013 NR genes, respectively. By using the randomly selected 123 regions from Refseq and NR genes, we normalized the population size and performed Fisher’s exact test. (C and D) Log_2_FC values of RNA-seq from DR genes and their H2H pair genes at 53 DR H2H loci in DMSO- or dTAG-13-treated cells are shown in a heatmap for the expression level (C) or a scatter plot for the relationship (D). The Spearman correlation coefficient and p value between DR H2H pairs is shown in (D). (E) The Pearson correlation coefficients of top 100 SETD1A co-dependent genes from DepMap database are shown. *PAF1* and *SPT5* are positively correlated. (F) Average ChIP-seq signal of PAF1, SPT5, and NELFe along DR (top panels) or NR (bottom panels) genes and flanking regions from DMSO-(black) or dTAG-13-treated (red or blue) FKBP-SETD1A cells at 24 h post-treatment are shown. (G–I) The ChIP-seq intensities (RPM) of NELFe (G) at the TSS ±1kb and PAF1 (H) and SPT5 (I) at the TES ±1 kb of DR genes in DMSO- or dTAG-13-treated FKBP-SETD1A cells at 24 h post-treatment are shown. (J) NELFe/PAF1 ratio at indicated gene loci in DMSO- or dTAG-13-treated cells at 24 h post-treatment are evaluated by ChIP-qPCR. Each ratio was calculated from the percentage of input of individual factors (see Figures S5F–S5H). Data are represented as mean ± SD. See also Figure S5.

To confirm the effect of SETD1A loss on RNA synthesis, we also performed nascent RNA-seq in dTAG-13-treated cells at 1.5 and 24.5 h post-dTAG-13 treatment and observed a significant reduction of newly synthesized mRNA from DR genes (Figures 5A and S5A). We previously identified that SETD1A binds with cyclin K, a co-factor of CDK9/12/13, which are all known as CTD kinases. CDK12 inhibition tends to suppress the expression of long genes, but DR and NR genes did not show any significant differences in gene size (Figure S5B).^17^ These data suggested that SETD1A might regulate elongation via a mechanism distinct from CDK12.

Genes for both DNA repair systems and mitochondrial respiration can be classified as H2H genes, which are regulated by bidirectional promoters.^25^ Interestingly, 43% of DR genes are classified as H2H genes with another gene located on the opposite strand within 1 kb upstream from the TSS (Figure 5B). H2H pairs tend to show similar expression patterns because of the shared promoter activity, but DR H2H pairs showed divergent gene expression after dTAG-13 treatment (Figures 5C and 5D).^26,27^ SETD1A localization just downstream from the TSS, the accumulation of Ser5P RNAPII, and the retardation of strand-specific RNAPII activity suggests an increase in promoter-proximal pausing of RNAPII at DR loci in a strand-specific manner. Paused RNAPII is modulated by the PAF1 complex, the negative elongation factor (NELF) complex, and the 5,6-dichloro-1-β-d-ribofuranosylbenzimidazole (DRB) sensitivity-inducing factor (DSIF) complex containing SPT5.^14^ In the DepMap database, SETD1A shows top co-dependencies with both PAF1 and SPT5, suggesting its role as a positive regulator of transcriptional elongation (Figure 5E). Although chromatin distributions of both PAF1 and SPT5 were not changed in dTAG-13-treated FKBP-SETD1A cells, NELFe, a subunit of the NELF complex, was significantly increased at the TSS of DR genes (Figures 5F, 5G, and S5C–S5E). In addition, both PAF1 and SPT5 were significantly decreased at the TES of DR genes (Figures 5H, 5I, and S5F). We also monitored the distributions of these factors at TSS of *GAPDH* (an NR gene), *HBB* (a non-SETD1A binding gene), and *COX15* (a DR gene) as well as the gene body of *COX15* by ChIP-qPCR (Figures S5G–S5I). High NELFe/PAF1 and NELFe/SPT5 ratios were only observed at *COX15* and were significantly increased by SETD1A depletion (Figures 5J and S5J). Taken together, our data demonstrate that SETD1A is required as a positive elongation regulator for the removal of the NELF complex followed by the release of paused RNAPII at H2H genes.

### Non-enzymatic role of SETD1A via cyclin K supports transcriptional activation and mitochondrial respiration in leukemia

**Figure 6.**
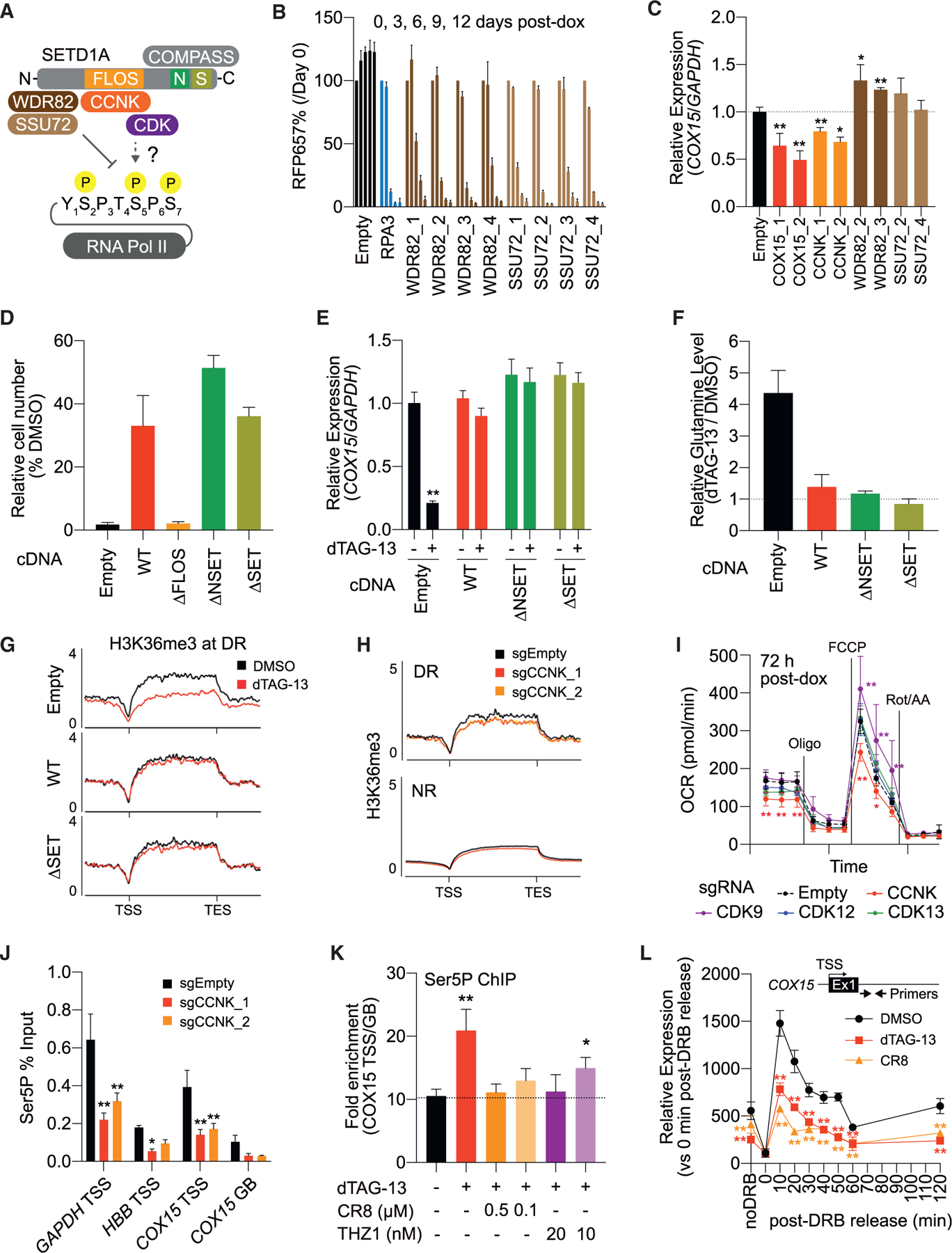
The non-enzymatic role of SETD1A via cyclin K is essential for the heme biosynthesis pathway and mitochondrial respiration in leukemia cells (A) Schematic model of SETD1A-dependent Ser5P regulation. FLOS, NSET (N), and SET (S) domains on SETD1A and their binding partners are shown. WDR82 binds Ser5P phosphatase SSU72, and cyclin K binds Ser5P kinases. The direct regulation of Ser5P via the SETD1A-cyclin K axis is unclear. (B) sgRNAs for WDR82 and SSU72 were transfected into iCas9-expressing MOLM-13 leukemia cells. Representative data from 2 independent experiments with 3 biological replicates are shown. (C) qRT-PCR analysis of *COX15* in the indicated sgRNA-expressing leukemia cells was performed at 4 days post-dox. Data from 3 independent experiments with 3 biological replicates are shown. (D) Human SETD1A deletion mutant-expressing FKBP-SETD1A cells were treated with DMSO or dTAG-13, and cell counts were performed at 6 days post-treatment. (E)*COX15* transcriptional level in SETD1A deletion mutant expressing FKBP-SETD1A cells was monitored by qRT-PCR at 24 h post-dTAG-13 treatment. (F) Relative Gln levels in dTAG-13-treated, SETD1A-rescued FKBP-SETD1A cells were analyzed at 24 h post-dTAG-13 treatment. (G) H3K36me3 levels at DR gene loci in SETD1A deletion mutant-expressing FKBP-SETD1A cells was quantified by ChIP-seq. (H) Average ChIP-seq signals of H3K36me3 level at DR and NR gene loci in cyclin K (CCNK)-knockout cells were evaluated at 4 days post-dox treatment (I) OCR in cyclin K/CDK9/12/13-knockout cells was evaluated by flux analyzer at 3 days post-dox treatment. Three to 4 independent sgRNAs were used for each molecule. Representative data from 2 independent experiments with 5 biological replicates are shown. (J) ChIP was performed with Ser5P antibody in cyclin K-knockout cells, and immunoprecipitated DNA was quantified by qPCR with specific primers for indicated target loci. (K) ChIP was performed with Ser5P antibody in dTAG-13/CR8/THZ1-treated FKBP-SETD1A cells at 24 h post-treatment, and immunoprecipitated DNA was quantified by qPCR with specific primers for *COX15* locus. Data from 3 biological replicates are shown. (L) DRB-release assay was performed in dTAG-13-or CR8-treated FKBP-SETD1A cells at 24 h post-treatment. Representative data from 2 independent experiments with 5 biological replicates are shown. Data are represented as mean ± SD. See also Figure S6.

SETD1A consists of three major domains including the N-terminal RNA recognition motif (RRM), central FLOS, and C-terminal NSET/SET domains. WDR82, the interaction partner of RRM, is known to bind with both Ser5P and CTD phosphatase (SSU72), but cyclin K, the interaction partner of FLOS, is known as a co-factor for CTD kinases (Figure 6A). Our data raised the possibility that the disruption of SETD1A may cause pausing of RNAPII in an SETD1A-complex-subunit-dependent manner. To evaluate the roles of WDR82 complexes and cyclin K complexes in human leukemia cells, we performed a CRISPR functional assay against these complex subunits and confirmed the strong dependency on WDR82, cyclin K, and their binding partners (Figures 6B and S6A). Despite the significant growth suppression in cyclin K-, WDR82-, and SSU72-knockout cells, only cyclin K knockout downregulated the expression of *COX15* (Figures 6B and 6C). To examine the role of FLOS and enzymatic domain in downstream *COX15* transcription, we established a cDNA rescue system in the PROTAC-based SETD1A-knockout model (Figure S6B). Consistent with our previous work, NSET/SET domain mutants rescued the cells from dTAG-13 treatment, but the FLOS domain mutant did not (Figure 6D). FLOS domain mutant-expressing cells also showed a similar decrease in expression and hypomethylation of H3K36me3 in the *COX15* gene compared with empty-vector-expressing cells (Figures S6C and S6D). Both the *COX15* expression and Gln levels were maintained in NSET/SET mutant-expressing rescued cells (Figures 6E and 6F). The hypomethylation of H3K36me3 was also rescued by NSET/SET domain mutant expression (Figures 6G and S6G). These results suggested that the SETD1A-cyclin K axis is the main upstream pathway for the *COX15* regulation. Supporting this model, cyclin K knockout results in H3K36me3 reduction on DR genes as well as the Gln accumulation (Figures 6H, S6H, and S6I). Cyclin K knockout, but not the individual CDK9/12/13 knockout, inhibited mitochondrial respiration (Figure 6I). Thus, the specificity under the SETD1A-cyclin K axis may not be dependent on a single CDK. Moreover, the cyclin K knockout showed reduction of both Ser5P and total RNAPII levels at the TSS (Figures 6J and S6J). These results suggest a requirement for cyclin K in SETD1A-loss-induced RNAPII pausing. To investigate the roles of CDK/cyclin K complex activities in SETD1A-loss-induced pausing, we treated cells with the CDK/cyclin K inhibitor CR8 or THZ1. The accumulation of both Ser5P RNAPII and NELFe at *COX15* locus were suppressed by both inhibitors (Figures 6K and S6K). Using a DRB release assay, we were able to confirm that there was a significant reduction of released RNAPII at the *COX15* locus after SETD1A loss and CR8 treatment (Figure 6L). The sustained release of RNAPII from the paused site, which was observed in control cells by 50 min after the DRB release, was not seen in dTAG-13-treated cells. Taken together, our study indicates that the aberrant function of the CDK/cyclin K complex following SETD1A disruption induced promoter-proximal pausing of RNAPII at H2H loci, which encode genes for DNA repair and mitochondrial metabolism.

## DISCUSSION

### Heme biosynthesis regulation in SETD1A-dependent cancer

**Figure 7.**
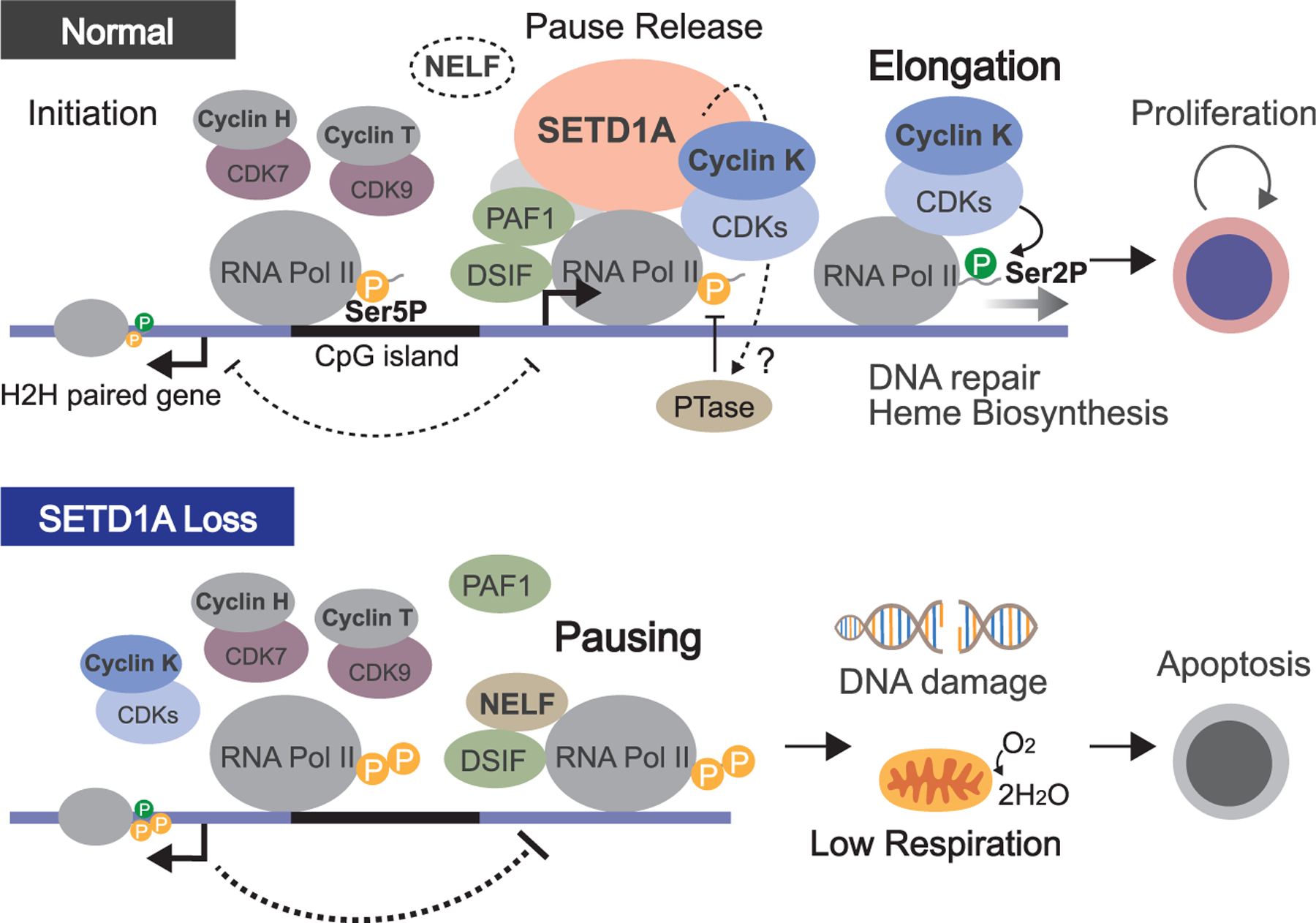
A model for the mechanism by which the non-enzymatic role of SETD1A promotes the transcriptional elongation and heme biosynthesis Transcriptional pause release is an important effector of non-enzymatic SETD1A function through cyclin K. In proliferating cells, SETD1A is required as a molecular switch to convert cyclin K functions from transcriptional initiation to productive elongation (top). The SETD1A-cyclin K complex with CDKs (CDK9/12/13) may promote substitution of NELF to PAF1 in the RNAPII complex, activation of Ser5P phosphatase (PTase), and elongation via Ser2 phosphorylation. The disruption of SETD1A stabilizes the Ser5 phosphorylated RNAPII and NELF complex, leading to pausing of transcriptional elongation at the TSS of genes associated with DNA repair and heme biosynthesis pathways at H2H loci (bottom). The elongation of genes on the opposite strand of the H2H pair or NR genes could be maintained by SETD1A-free elongation regulators. The genomic structure or transcription of the paired gene may also contribute to pausing stabilization of SETD1A downstream genes.

Our results revealed that SETD1A is essential for the maintenance of gene expression contributing the heme biosynthesis pathway (Figure 7). Upregulation of the heme biosynthesis pathway caused by an increase of UROD expression is observed in pediatric AML samples with high MYCN expression.^28^ A recent study indicated the useful application of the knockdown of the heme biosynthesis pathway against Bcl-2 inhibitor resistance in AML cells.^29^ In this study, AML cell lines showed strong sensitivity to *COX15*-targeting sgRNAs, unlike chronic myeloid leukemia (CML) and sarcoma cell lines. Therefore, dependency on the heme biosynthesis pathway would partly explain the effectiveness of SETD1A-targeting therapy, specifically in AML. One critical role of the heme biosynthesis pathway could be the maintenance of mitochondrial respiration. However, mitochondrial defects, besides the reduction of respiration, have not been observed in previous reports nor our study. These results suggest a specific role for the heme biosynthesis pathway in AML cell proliferation. In this study, we also identified the upregulation of Gln levels in SETD1A/cyclin K-deficient cells. Gln is an ingredient of heme biosynthesis and an important nutrient for cell proliferation. The inhibition of Gln uptake is currently being studied as a potential therapeutic tool for AML therapy.^30,31^ Therefore, a future combination therapy of SETD1A inhibition and Gln uptake inhibition could represent an approach for AML treatment.

SETD1A is frequently mutated in the brain of patients with neurogenerative disease,^32,33^ and the heterozygous mutation in mice recapitulates the schizophrenia-related phenotypes.^4,6^ These mutations are not only observed at the enzymatic domain but also at the FLOS domain.^32^ Understanding the molecular mechanisms behind the SETD1A-dependent heme biosynthesis pathway will offer insight into neuronal disorders and other diseases.

### SETD1A is a critical regulator for the CTD code

Our data show unexpected upregulation of Ser5P in SETD1A-deficient AML cells. Ser5 is a well-characterized CTD phosphorylation site that is phosphorylated by multiple CDKs, including CDK7/9/12/13. Together with the PAF complex, Ser5P promotes the recruitment of Set1, a SETD1A homolog, to the TSS in yeast.^34,35^ However, the molecular mechanism by which loss of SETD1A alters the phosphorylation of the CTD code was unclear.

Here, we only evaluated the role of SSU72, which is a CTD phosphatase that binds with the SETD1A subunit WDR82. A recent study identified the new CTD phosphatase activity on the integrator complex.^36^ Ser5-dephosphorylation-associated proteins (RPRD1A/RPRD1B) may also contribute to the SETD1A-dependent phenotypes.^37^ While there is a possibility that SETD1A may cooperate with multiple phosphatases, our data indicated the essential role of cyclin K in the upregulation of Ser5P by through the multiple CDKs. In addition to the cyclin K-dependent CDKs, CDK7 may partly contribute the accumulation of Ser5P since the treatment of THZ1, a covalent inhibitor of CDK7/12/13, suppressed the phosphorylation in SETD1A-knockout cells.^38^ We also observed the upregulation of the Ser5P/RNAPII ratio at the gene body of NR genes. Interestingly, the increased Ser5P in the TSS and gene body has also been reported in PAF1-deficient cells.^39^ In contrast, DR genes showed an unaffected level of Ser5P and a decrease of Ser2P at their gene body. These similarities and differences indicated that further studies on the SETD1A-PAF1-CDKs axis would be required to understand the regulation of CTD code as well as the regulation of specific genes followed by the SETD1A degradation.

Inhibition of CDKs by small-molecule compounds has already been achieved in many studies, and these inhibitors will be great opportunities for cancer therapy, but the specificity against CDK/cyclin K complexes and the locus dependency on chromatin remain unclear. Unlike the inhibition of cyclin K, inhibition of SETD1A suppressed the Ser2P and productive elongation without inhibiting the Ser5P (Figure 7). As we observed the locus-dependent transcriptional defect after SETD1A degradation, an SETD1A inhibitor will provide a more specific tool for cancer therapy than CDK kinase inhibitors by modulating the CTD code on cancer-associated genes.

### Possible evolutionary background of the RNAPII pause release via SETD1A

An interesting feature of the SETD1A-sensitive loci is that the encoded genes are regulated under the H2H promoters in a strand-specific manner. Previous works only focused on the H3K4 methyltransferase activity of Set1 as a downstream effector and did not examine whether Set1 or SETD1A itself is required for the pause release.^34,35^ The emerging nascent RNA is recognized by an SPT4/SPT5 complex called DSIF, then the NELF complex binds the RNAPII/DSIF complex and pauses the weakly activated RNAPII at the promoter-proximal region.^40^ NELF homologs have been identified in metazoans but not in yeast and plants; therefore, NELF would contribute the specific roles in the transcriptional regulation in metazoans.^40^ The SETD1A FLOS domain has also not been identified in yeast and evolved from vertebrates, therefore the SETD1A-NELF axis would play a specialized action for the RNAPII regulation in vertebrates.

Genes under bidirectional promoters are thought to have biological significance in vertebrates, whose genomes are more dispersed than the densely organized genomes of yeast and lower organisms. One of the common features of the bidirectional loci is the presence of a CpG island (CGI) on the promoter.^27^ Non-methylated CGI in active loci is important for the recruitment of CXXC domain-containing protein, such as CXXC1, a binding partner of SETD1A/B.^41^ In contrast, CXXC1 binds to the NSET domain of SETD1A, but the loss of elongation was fully restored in the NSET domain mutant. Further study will be necessary to demonstrate whether the CGI recognition of SETD1A is required for the regulation of H2H gene activation.

Genes under bidirectional promoters are required for DNA repair, replication, and mitochondria function. Thus, a synchronized regulation for these genes could be important for cell division with continuous cell cycling.^25,27^ The pause release of RNAPII also has an important role in synchronous patterns of gene activation.^42^ The evolution of the pause release system in H2H genes might have coincided with the molecular evolutions of the SETD1A-NELF axis to enable more precise and synchronized gene regulation in vertebrates. Taken together, targeting of this pathway with a SETD1A inhibitor will provide therapeutic benefits for mammalian cancers and diseases.

### Limitations of the study

We used MLL-r leukemia cell lines for the SETD1A PROTAC model in this study but did not utilize them for *in vivo* assays. We evaluated these cells under normal cell culture conditions; therefore, this study may not reflect *in vivo* responses under the specific microenvironments.

## STAR★METHODS

### RESOURCE AVAILABILITY

#### Lead contact

Further information and requests for resources and reagents should be directed to and will be fulfilled by the lead contact, Takayuki Hoshii (hoshiit@chiba-u.jp).

#### Materials availability

All unique/stable reagents generated in this study are available from the lead contact with a completed Material Transfer Agreement.

#### Data and code availability

- The accession number for the RNA-seq and ChIP-seq data reported in this paper is NCBI GEO: GSE189894. The MS raw data and result files have been deposited in the ProteomeXchange Consortium: PXD029667 via the jPOST partner repository: JPST001381.^1^ The metabolomics data is available via the Metabolomics Workbench: PR001489.^2^
- This paper does not report original code.
- Any additional information required to reanalyze the data reported in this paper is available from the lead contact upon request.

### EXPERIMENTAL MODEL AND SUBJECT DETAILS

#### Cell lines

293T, MV4–11, U937, K562, A673, RH30 human cell lines were obtained from ATCC. MOLM-13 human cell line was obtained from DSMZ. Plat-A, Plat-E cell lines which were both based on the 293T cell line were obtained from Dr. Toshio Kitamura. 293T, A673, Plat-A, Plat-E cell lines were maintained in DMEM medium containing 10% fetal bovine serum (FBS) and 1% penicillin-streptomycin. U937, MOLM-13 and RH30 cell lines were maintained in RPMI1640 medium containing 10% FBS and 1% penicillin-streptomycin. MV4–11 and K562 cell lines were maintained in IMDM medium containing 10% FBS and 1% penicillin-streptomycin. All cell lines were authenticated using STR profiling with a GenePrint 24 system (Promega). Murine *Setd1a*^*flox/flox*^;*CreER* MLL-r leukemia cells were established previously.^7^ Murine MLL-r leukemia cells were maintained in RPMI1640 medium containing 10 ng/mL rmIL3, 10% FBS and 1% penicillin-streptomycin. Cells were maintained in a humidified incubator at 37°C, 5% CO_2_.

### METHOD DETAILS

#### Drug treatment

Doxycycline was prepared to a stock solution in 1 mg/mL in PBS and used as 1:1000 dilution.

dTAG-13, CR8 or THZ1 was prepared to a stock solution in 1mM in DMSO. For dTAG-13, we use 500 nM dTAG-13 for a final concentration if we didn’t mention the concentration in each data. For CR8 or THZ1, we use in 0.5/0.1 μM or 20/10 nM, respectively.

#### Establishment of dTAG-SETD1A system

Human SETD1A (NM_014712) cDNA was cloned into pLEX_305-N-dTAG or pLEX_350-C-dTAG plasmid vector. Expression vector was transduced into mouse *Setd1a*^*flox/flox*^;*CreER*^*T2*^;*MLL-AF9* leukemia cells or Cas9-expressing human leukemia cells.^7^ Transduced cells were selected by 2 μg/mL puromycin for a week. To remove the endogenous SETD1A expression, 1 μg/mL tamoxifen was administrated into cell culture medium for mouse leukemia cells. For human cell lines, SETD1A sgRNA for intron-exon junction was transduced into the puromycin-resistant human leukemia cells and GFP-positive sgRNA-expressing cells were collected by cell sorter SH800S (SONY). Single cell clones from these cells were established by limiting dilution. All single cell clones were genotyped by PCR, and each indel sequence was determined by sanger sequencing. Protein expression from exogenous FKBP^F36V^-tagged-SETD1A construct or the endogenous SETD1A allele were monitored with anti-HA antibody or anti-SETD1A antibody on western blot analyses, respectively.

#### cDNA expression in leukemia cells

EGFP-OMP25-expressing vector was transduced into *Setd1a*^*flox/flox*^;*CreER* MLL-AF9 leukemic cells by retrovirus which is generated in Plat-E cells. EGFP-OMP25 subcellular localization in leukemic cells was monitored by FLUOVIEW FV10i (Olympus). Signal intensities of EGFP-OMP25 was quantified by CytoFLEX flow cytometry (Beckman coulter). For cDNA rescue experiment, SETD1A-expressing vector with hygromycin-resistant gene was transduced into FKBP-SETD1A leukemic cells by retrovirus which is generated in Plat-A cells. Transduced cells were selected by 1 mg/mL hygromycin for a week. For the SETD1A mutant constructs, FLOS (387–586 aa), NSET (1425–1562 aa) or SET (1568–1691 aa) domains were deleted from SETD1A-expression vector by inverse PCR method.

#### Western blotting

Lysate was mixed with 43 SDS Sample Buffer and beta-mercaptoethanol, and proteins were denatured by boiling for 5 min. Denatured proteins were separated on a 5–20% SuperSep TM Ace gel (Wako) in 25 mM Tris/192 mM Glycine/0.1% SDS Running Buffer and transferred to a PVDF membrane (Merck Millipore) in 25 mM Tris/192mM Glycine/20% Methanol Transfer Buffer with Mini Trans-Blot Cell (Bio-Rad). Blots were blocked with 5% skim milk in TBS-T for 30 min, and incubated with primary antibodies (which are listed in the key resource table) at 4°C overnight. Immunocomplexes were labeled by HRP-conjugated anti-mouse IgG or anti-rabbit IgG and visualized using Amersham ECL Prime (Cytiva). The signals were deteceted with ChemiDoc Touch MP (Bio-Rad).

#### RNA analyses

Total RNA was purified with RNeasy Mini kit with DNase set (Qiagen). For RT-qPCR, cDNA was synthesized with ReverTra Ace qPCR RT Master Mix (TOYOBO). cDNA fragments were quantified by a SYBR green qPCR with gene specific primers and Taq DNA polymerase (NEB) with CFX96 Touch real-time PCR detection system (Bio-Rad). For Nascent RNA analysis, 1 × 10^6^ cells were treated with 500 nM dTAG-13 for 1 h or 24h. 500 nM EU was added and incubated for additional 0.5 h. After the EU incorporation, total RNA was extracted with Direct-zol RNA miniprep kit (Zymo Research) at 1.5 h or 24.5 h post-dTAG-13 treatment. RiboMinus Human/Mouse Transcriptome Isolation Kit (ThermoFisher) was used for the removal of rRNA, and then EU-labelled RNA was enriched by using Click-iT Nascent RNA Capture kit (ThermoFisher). Libraries for RNA-seq were prepared by using the TruSeq Stranded mRNA Sample Prep Kit (Illumina). The DNA library was validated using TapeStation (Agilent Technologies) and was quantified using a QuantiFluor dsDNA system (Promega) and a Quantus Fluorometer (Promega). Libraries were pooled and sequenced on Illumina HiSeq 1500 or NextSeq 500. Data were analyzed by using HISAT2 and Cufflinks, or workflows on Basepair (https://www.basepairtech.com). List of interest genes were analyzed by Enrichr tool (https://amp.pharm.mssm.edu/Enrichr/).

#### ChIP analyses

MOLM-13 leukemia cells were fixed with 2 mM DSG for 30 min followed by 1% formaldehyde for 10 min and treated with 0.125 M Glycine for 5 min. Fixed cells were washed twice with cold PBS. Washed cells were re-suspended in ChIP Lysis Buffer (50 mM HEPES pH8, 140 mM NaCl, 1 mM EDTA, 10% Glycerol, 0.5% NP-40, 0.25% Triton-X100, 13 protease inhibitor cocktail) and centrifuged at 3,200 rpm for 5 min at 4°C to collect the nuclei pellet. The pellet was washed with ChIP Wash Buffer (10 mM Tris-HCl, 200 mM NaCl, 1 mM EDTA), and re-suspended in ChIP Shearing Buffer (0.1% SDS, 1 mM EDTA, 10 mM Tris-HCl, 13 protease inhibitor cocktail) and then frozen at −80°C. Nuclear samples were shredded using Picoruptor (Diagenode) and pre-cleared with Dynabeads protein G (Thermo Fisher Scientific) for 60 min at 4°C with gentle rotation. Appropriate amounts of antibodies were added into the chromatin and incubated overnight at 4°C. The immune complex was collected with Dynabeads protein G and washed sequentially in the Low Salt Wash Buffer (20 mM Tris pH8, 150 mM NaCl, 0.1% SDS, 1% Triton-X100, 2 mM EDTA), the High Salt Wash Buffer (20 mM Tris pH8, 500 mM NaCl, 0.1% SDS, 1% Triton-X100, 2 mM EDTA), the LiCl Wash Buffer (10 mM Tris pH8, 250 mM LiCl, 1% NP-40, 1% Sodium Dexyxholate, 1 mM EDTA) and TE. Chromatin was eluted with Elution Buffer (1% SDS, 20 mM Tris-HCl, 10 mM EDTA) containing RNaseA, and then reverse cross-linked with proteinase K at 65°C for overnight. DNA was purified with a PCR purification kit (Qiagen) and was quantified using a QuantiFluor dsDNA system (Promega) and a Quantus Fluorometer (Promega). ChIP DNA was quantified by SYBR Green Real-time PCR with the specific primers and NEB taq polymerase (NEB) using CFX96 Real-Time PCR detection system (Bio-rad). Libraries were prepared and sequenced as described above. Data were mapped to the University of California Santa Cruz human genome assembly (hg19) using Bowtie2, and then duplicated reads were removed by Picard tools. Peak calling was performed using HOMER software. Heatmaps or histograms of ChIP-seq data were generated by using EaSeq software (http://easeq.net). Gene annotation to ChIP-seq peaks were performed with the Genomic Regions Enrichment of Annotations Tool (GREAT) using the basal plus extension method.

### CRISPR

Doxycycline-inducible Cas9-expressing human MOLM-13 (MOLM-13iCas9), U937 and K562 leukemia cells and constitutive Cas9-expressing A673 and RH30 sarcoma cells were established previously.^7^ sgRNA vectors with a GFP or tagRFP657 reporter were constructed and used for competitive growth assay (Table S1). At least four sgRNA oligos for each target were selected from CRISPick at Broad institute (https://broad.io/crispick). The empty vector backbone was used for the negative control, and the human *RPA3*-targeting sgRNA was used for the positive control in competitive growth assay. The SETD1A intron 7-exon 8 junction-targeting sgRNA was used for the deletion of endogenous *SETD1A* genes in human leukemia cells (Table S1). In competitive growth assay with these sgRNA constructs, vectors were transduced to the iCas9 leukemia cells or Cas9 sarcoma cells with lentivirus. The expression of fluorescent proteins was confirmed at 3 days post-infection by using CytoFLEX flow cytometry (Beckman coulter), then doxycycline was added in the culture medium. In doxycycline inducible system, the first day for the doxycycline administration was set as day 0 for the competitive growth assay, and the percentage of fluorescent protein-positive population was measured every 3 days. Percentage of sgRNA/RFP657-expressing cells were normalized with the percentage at 0 day post-dox For the sarcoma cell lines, the percentage of fluorescent protein-positive population was confirmed at 3 days post-infection, and monitored every 6 days.

#### Detection of mitochondria respiration and glycolysis

Oxygen consumption rate and extracellular acidification rate of leukemia cells were measured by Seahorse XF Analyzer (Agilent) with XF Cell MitoStress Test Kit (Agilent) and XF Glycolysis Stress Test Kit (Agilent), respectively. Cell culture plate for XFe96 was coated with Cell-Tak cell tissue adhesive (Corning) and 1 × 10^5^ leukemia cells were plated before the measurement. 10 μM of Oligo (Oligomycin: ATP synthase inhibitor), 20 μM of FCCP (OXPHOS uncoupler) and 5μM of Rot/AA (Rotenone/antimycin A: inhibitors of mitochondrial electron transport chain) were used for each well in Mito stress Test Kit at indicated time points. 20 μL of 100 mM Glucose, 22 μL of 10 μM Oligomycin and 25 μL of 500 mM 2-DG were used for each well in Glycolysis Stress Test Kit. We set 3 min for mixture and 3 min for measurement for each cycle, and actual recorded time point were shown on graphs.

#### Mitochondria isolation and proteomics

Human Mitochondria Isolation Kit (Miltenyi Biotec) was used for the mitochondria isolation from MOLM-13 leukemia cells. 1 × 10^7^ cells were harvested and washed with cold-PBS and resuspended in 1 mL ice-cold Lysis Buffer. Cells were homogenized by using a 29 G syringe at 20 strokes. To remove nuclei, cell lysates were centrifuge at 1,000 × g for 5 min at 4°C, then transfer the 1 mL of supernatants into new 15 mL tubes. 9 mL of ice-cold 1 × Separation Buffer and 50 µL of anti-TOM22 microbeads were added and incubated for 1 h at 4°C with gentle shaking. Microbeads were separated with MACS separator with LS columns. After washing step, we flushed out the magnetically labeled mitochondria and washed once with ice-cold PBS. Mitochondria pellets were frozen at liquid nitrogen and stored at –80°C. Proteins were extracted with 100 mM triethyl ammonium bicarbonate containing 12 mM sodium deoxycholate (SDC) and 12 mM sodium lauroyl sarcosinate (SLS), and were reduced using 10 mM dithiothreitol for 30 min followed by alkylation with 50 mM iodoacetamide for 30 min. Tryptic digested peptides were labeled with the Tandem Mass Tag reagents and combined samples. After removal of SDC and SLS by a phase transfer,^51,52^ peptides were separated by a High pH Reversed-Phase Peptide Fractionation Kit (Thermo Scientific). NanoLC-MS/MS were conducted using an Orbitrap Fusion Tribrid mass spectrometer (Thermo Scientific) and an Easy nLC-1000 UHPLC (Thermo Scientific) equipped with a nanoHPLC capillary column (Nikkyo Technos, Tokyo, Japan). MS data were subjected to a search against the Uniprot Human database with MaxQuant version 1.6.17.0.^53^

#### Metabolome analysis

Metabolomic profiling was conducted for triplicated DMSO/dTAG-13 treated leukemia cell extracts via capillary electrophoresis time-of-flight mass spectrometry (CE-TOFMS) by using Agilent 7100 CE capillary electrophoresis (Agilent Technologies), the Agilent 6230 LC/MSD TOF system (Agilent Technologies), an Agilent1100 series binary HPLC pump, and the G1603A Agilent CE-MS adapter- and G1607A Agilent CE-ESI-MS sprayer kit.^54,55^ For anionic metabolite analysis, the original Agilent stainless ESI needle was replaced with the Agilent G7100–60041 platinum ESI needle. System control and data acquisition were performed by Agilent MassHunter Workstation, and data analysis was done by Keio MasterHands software. For cationic metabolite analysis, separations were carried out in a fused silica capillary (50 μm i.d. × 100 cm total length) filled with 1 M formic acid as the electrolyte. Approximately 5 nL of sample solution were injected at 50 mbar for 5 s and 30 kV of voltage was applied. The capillary temperature was maintained at 20°C and the sample tray was cooled below 5°C. Methanol-water (50% v/v) containing 0.01 μM Hexakis(2,2-difluoroethoxy)phosphazene was delivered as the sheath liquid at 10 μL/min. Electrospray ionization (ESI)-time-of-flight mass spectrometry (TOFMS) was conducted in the positive ion mode and the capillary voltage was set at 4,000V. A flow rate of heated dry nitrogen gas (heater temperature 300°C) was maintained at 7 psig. In TOFMS, the fragmentor-, skimmer-, and Oct RFV voltage was set at 75 V, 50 V, and 500V, respectively. Automatic recalibration of each acquired spectrum was performed using reference masses of reference standards. The ^13^C isotopic ion of a protonated methanol dimer ([2MeOH + H]^+^, m/z 66.0631) and Hexakis(2,2-difluoroethoxy)phosphazene ([M + H]^+^, m/z 622.0290) provided the lock mass for exact mass measurements. For anionic metabolite analysis, a commercially available COSMO(+) (chemically coated with cationic polymer) capillary (50 μm i.d. × 105 cm total length) (Nacalai Tesque, Kyoto, Japan) was used with a 50 mM ammonium acetate solution (pH 8.5) as the electrolyte. Sample solution (30 nL) was injected at 50 mbar for 30 s and –30 kV of voltage was applied. Ammonium acetate (5 mM) in 50% methanol-water (v/v) containing 0.01 μM Hexakis(2,2-difluoroethoxy)phosphazene was delivered as the sheath liquid at 10 μL/min. ESI-TOFMS was conducted in the negative ion mode; the capillary voltage was set at 3,500 V. For TOFMS, the fragmentor-, skimmer-, and Oct RFV voltage was set at 100 V, 50 V, and 500 V, respectively. Automatic recalibration of each acquired spectrum was performed using reference masses of reference standards, i.e., ^13^C isotopic ion of deprotonated acetic acid dimer ([2CH_3_COOH-H]^–^, m/z 120.0384), and Hexakis + deprotonated acetic acid (m/z 680.03554) provided the lock mass for exact mass measurements.

#### Glutamine/glutamate assay and ATP assay

To determine the glutamine and glutamate level in cells, we used Glutamine/Glutamate-Glo assay kit (Promega). Extracts from 20,000 cells were used for each wells. For ATP assay, we used CellTiter-Glo 2.0 cell viability assay kit (Promega). 10,000 cells were seeded onto white 96-well plate and equilibrated at room temperature for 30 min 100 μL of detection reagent were added and incubated for 10 min. Luciferase signals from both assays were measured by Synergy LX multi-mode microplate reader (BioTek).

#### Cell cycle and apoptosis assay

Cell cycle was measured with Click-iT EdU Flow Cytometry Assay Kit (ThermoFisher). Cells were treated with 10 μM EdU containing medium for 2 h and fixed with fixative buffer in the kit. Incorporated EdU was labelled with Alexa647-conjugaed azide. Stained cells were counter stained with 7AAD. For Apoptosis assay, cells were washed with 1× annexin V binding buffer (abcam), and then incubated with Annexin V-APC containing buffer at room temperature for 10 min. Cells were washed with 1× annexin V binding buffer and stained with 7AAD. Stained cells were analyzed with CytoFLEX flow cytometer (Beckman Coulter).

#### DRB-release assay

Cells were treated with 100 μM DRB containing medium for 3.5 h, washed with PBS twice, and then cultured in the normal medium for a given period. The samples at 0 min after DRB release were washed with 100 μM DRB containing PBS twice to prevent the unintentional RNAP2 re-activation during the washing period. The purification of total RNA and cDNA synthesis were performed as described above. Pre-mRNA production from released RNAP2 was quantified with SYBR Green Real-time PCR with the specific primer for 1^st^ intron of COX15 locus (Table S2) using CFX96 Real-Time PCR detection system (Bio-rad). Pre-mRNA expression levels were normalized with the background expression level in the samples at 0 min after DRB release.

### QUANTIFICATION AND STATISTICAL ANALYSIS

Error bars in all of the data represent a standard deviation. For statistical comparison, we performed a Student’s *t*-test or one-way ANOVA followed by Tukey’s test. Simple linear regression was used to compare the relationship between two quantitative values in Figures 4C and 4D, 5D, S4D and S4E. We identified 1921 H2H pairs that have overlapped promoter (1 kb up from TSS) from the hg19 reference sequence encoding 14433 and 13828 TSS regions from plus and minus strand, respectively (Figure 5B). The enrichment of H2H locus in DR group were evaluated with Fisher’s exact test. To normalize the difference on population size among Refseq, NR and DR, we randomly and repeatedly (5 times) picked up 123 regions from Refseq and NR by Select random lines, and then average numbers were used. Data with statistical significance (*p < 0.05, **p < 0.01) or no significance (ns; p > 0.05) are shown in figures. Statistical analyses were performed using Prism 9 software (GraphPad).

## Supplementary Material

1

## Figures and Tables

**Table:** KEY RESOURCES TABLE

REAGENT or RESOURCE	SOURCE	IDENTIFIER
Antibodies		

Rabbit monoclonal anti-HA	Cell Signaling	Cat#3724, RRID: AB_1549585
Rabbit monoclonal anti-SETD1A	Cell Signaling	Cat#61702, RRID: AB_2799614
Rabbit monoclonal anti-GAPDH	Cell Signaling	Cat#2118, RRID: AB_561053
Rabbit monoclonal anti-H3K4me1	Cell Signaling	Cat#5326, RRID: AB_10695148
Rabbit monoclonal anti-H3K4me3	Active Motif	Cat#39159, RRID: AB_2615077
Rabbit monoclonal anti-H3K27Ac	Active Motif	Cat#91193, RRID: AB_2793797
Rabbit monoclonal anti-H3K36me3	Abcam	Cat#9050, RRID: AB_306966
Rabbit monoclonal anti-H3K79me2	Abcam	Cat#3594, RRID: AB_303937
Rat monoclonal anti-phospho-Rpb1 CTD (Tyr1)	Active Motif	Cat#61383, RRID: AB_2793613 Rabbit monoclonal anti-phospho-Rpb1 CTD (Ser2)
Rabbit polyclonal anti-PAF1	Abcam	Cat#ab20662, RRID: AB_2159769
Rat monoclonal anti-SPT5	Active Motif	Cat#65689
Rabbit monoclonal anti-NELFe	Abcam	Cat#ab170104, RRID: AB_2827280
Rat monoclonal anti-phospho-Rpb1 CTD (Tyr1)	Active Motif	Cat#61383, RRID: AB_2793613 Rabbit monoclonal anti-phospho-Rpb1 CTD (Ser2)
Rabbit polyclonal anti-PAF1	Abcam	Cat#ab20662, RRID: AB_2159769
Rat monoclonal anti-SPT5	Active Motif	Cat#65689
Rabbit monoclonal anti-NELFe	Abcam	Cat#ab170104, RRID: AB_2827280

Chemicals, peptides, and recombinant proteins		

dTAG-13	Tocris Bioscience	Cat#6605
Doxycycline	Merck	Cat#D9891

Deposited Data		

Raw and analyzed data of RNA-seq and ChIP-seq	This paper	GEO: GSE189894
Raw data of Mass spectrometry analysis	This paper	ProteomeXchange:
Analyzed data of metabolome analysis	This paper	Metabolomics Workbench: PR001489

Experimental models: Cell lines		

Human: 293T cells	ATCC	CRL-3216
Human: Plat-A cells	Morita et al. 2000^43^	N/A
Human: MV4–11 cells	ATCC	CRL-9591
Human: U937 cells	ATCC	CRL-1593.2
Human: K562 cells	ATCC	CCL-243
Human: A673 cells	ATCC	CRL-1598
Human: SJCRH30 (RH30) cells	ATCC	CRL-2061
Human: MOLM-13 cells	DSMZ	ACC-554
Mouse: Setd1a^flox/flox^;CreER^T2^;MLL-AF9 leukemia cells	Hoshii et al. 2018^7^	N/A

Oligonucleotides		

sgRNA (see Table S1)	This paper	N/A
PCR Primers (see Table S2)	This paper	N/A

Recombinant DNA		

Plasmid: pLKO5.sgRNA.EFS.GFP	Heckl et al. 2014^44^	Addgene #57822
Plasmid: pLKO5.sgRNA.EFS.tRFP657	Heckl et al. 2014^44^	Addgene #57824
Plasmid: pMD2.G	Laboratory of Didier Trono	Addgene #12259
Plasmid: psPAX2	Laboratory of Didier Trono	Addgene #12260
Plasmid: pCMV6-hSETD1A(NM_014712)-Myc-DDK	Origene	Cat#RC214996
Plasmid: pMXs-3XHA-EGFP-OMP25	Chen et al. 2016^45^	Addgene #83356

Software and algorithms		

Prism 9	GraphPad software	N/A
EaSeq	Lerdrup et al. 2016^46^	http://easeq.net
Analysis of RNA-seq data	Basepair software	https://www.basepairtech.com
HISAT2 (2.1.0)	Kim et al. 2019^47^	http://daehwankimlab.github.io/hisat2/
Cufflinks (2.2.1)	Trapnell et al. 2010^48^	http://cole-trapnell-lab.github.io/cufflinks/
Bowtie2 (2.3.3.1)	Langmead and Salzberg, 2012^49^	http://bowtie-bio.sourceforge.net/bowtie2/index.shtml
Picard (2.18.2)	Broad Institute	https://broadinstitute.github.io/picard/
HOMER (4.10)	Heinz et al. 2010^50^	http://homer.ucsd.edu/homer/

## Highlights

- H3K4 methyltransferase SETD1A regulates heme biosynthesis pathway genes
- SETD1A is required for mitochondrial respiration in acute myeloid leukemia cells
- Loss of SETD1A increases Ser5P RNA polymerase II at the transcription start sites
- A non-enzymatic domain of SETD1A modulates transcriptional pause release
