# Supplementary material for: SETD1A regulates transcriptional pause release of heme biosynthesis genes in leukemia
Source: Cell Rep. Author manuscript; Available in PMC 2022 Dec 21. (PMC9771694; doi:10.1016/j.celrep.2022.111727)
Supplement: 1 [file NIHMS1854083-supplement-1.pdf]

Cell Reports, Volume *41*

Supplemental information

SETD1A regulates transcriptional pause release of heme biosynthesis genes in leukemia

Takayuki Hoshii, Sarah Perlee, Sota Kikuchi, Bahityar Rahmutulla, Masaki Fukuyo, Takeshi Masuda, Sumio Ohtsuki, Tomoyoshi Soga, Behnam Nabet, and Atsushi Kaneda

Supplemental information

SETD1A regulates transcriptional pause release of heme biosynthesis genes in leukemia

Takayuki Hoshii, Sarah Perlee, Sota Kikuchi, Bahityar Rahmutulla, Masaki Fukuyo, Takeshi Masuda, Sumio Ohtsuki, Tomoyoshi Soga, Behnam Nabet, Atsushi Kaneda.

Supporting Information

This SI file includes Figures. S1 to S6 and Tables. S1 to S2.

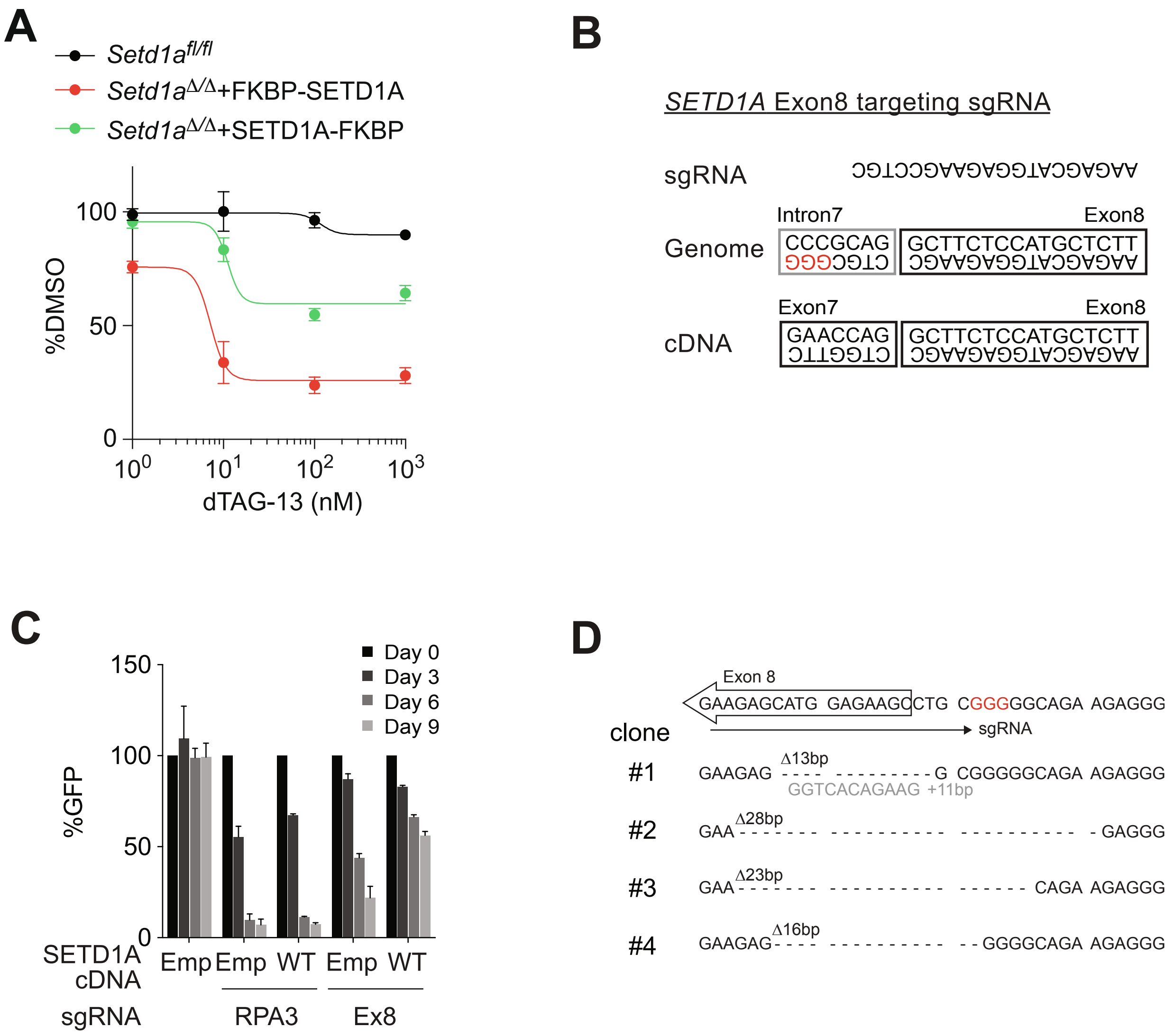
A
Setd1a fl/fl
Setd1a Δ/Δ+FKBP-SETD1A
Setd1a Δ/Δ+SETD1A-FKBP
%DMSO
100
50
0
10^0
10^1
10^2
10^3
dTAG-13 (nM)
B
SETD1A Exon8 targeting sgRNA
sgRNA
Genome
Intron7
Exon8
CCCGCAG
GCTTCTCCATGCTCTT
cDNA
Exon7
Exon8
GAACCAG
GCTTCTCCATGCTCTT
C
%GFP
150
100
50
0
Day 0
Day 3
Day 6
Day 9
SETD1A cDNA
Emp
Emp
WT
Emp
WT
sgRNA
RPA3
Ex8
D
Exon 8
GAAGAGCATG GAGAAGCCTG CGGGGGCAGA AGAGGG
sgRNA
clone
#1
GAAGAG Δ13bp G CGGGGGCAGA AGAGGG
GGTCACAGAAG +11bp
#2
GAA Δ28bp GAGGG
#3
GAA Δ23bp CAGA AGAGGG
#4
GAAGAG Δ16bp GGGGCAGA AGAGGG

Figure S1. SETD1A degradation induces cell growth arrest in both mouse and human MLL-r leukemia cells, Related to Figure 1.

A. *Setd1a*$^{fl/fl}$, FKBP^{F36V}-HA-SETD1A-expressing *Setd1a*$^{\Delta/\Delta}$;CreER (FKBP-SETD1A), or SETD1A-FKBP^{F36V}-HA-expressing *Setd1a*$^{\Delta/\Delta}$;CreER (SETD1A-FKBP) MLL-AF9 leukemia cells were treated in 0 to 1000 nM dTAG-13, then cell number was counted at 6 days post-treatment.

B. The DNA sequences of SETD1A sgRNA in this study, the junction between intron 7 and exon 8 of human SETD1A gene, and the junction between exon 7 and exon 8 of human SETD1A cDNA are shown.

C. SETD1A exon 8 targeting sgRNA was transfected into the FKBP-SETD1A-expressing MOLM-13;Cas9-Blast cells, and the percentage of GFP-positive cells were monitored at every 3 days by flow cytometry.

D. The indel mutations in 4 independent rescued cells with FKBP-SETD1A were examined by the single cell cloning followed by the DNA sequencing.

Data are represented as mean ± SD.

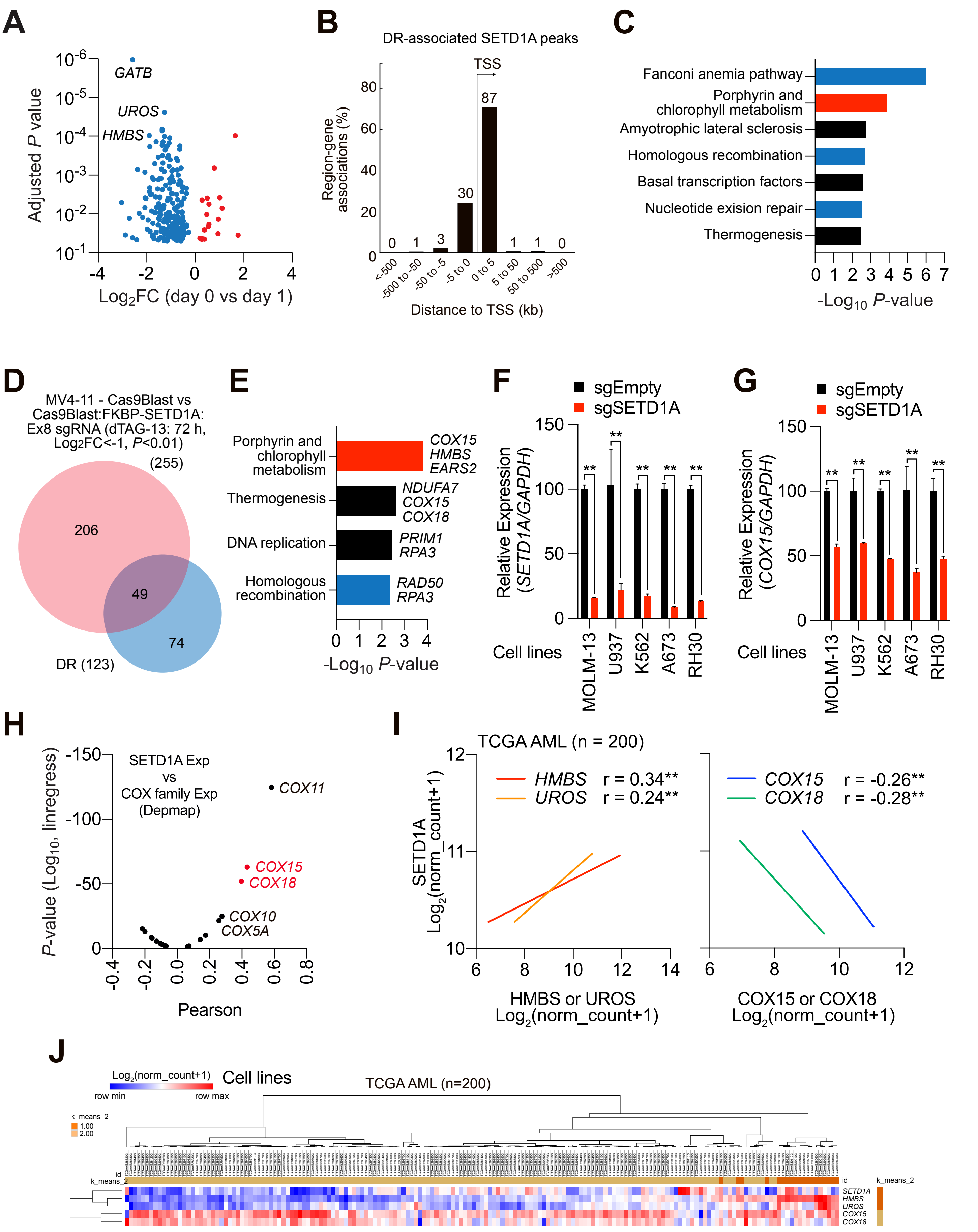

Hoshii T - Figure S2

Figure S2. The heme biosynthesis pathway and DNA repair pathway are dependent on the SETD1A in leukemia cells, Related to Figure 2.

A. RNA-seq analysis against DMSO or dTAG-13 treated FKBP-SETD1A cells at day 1.

B. Distribution of FKBP-SETD1A peaks that annotated to DR genes was shown. 123 DR peaks were re-annotated to single nearest genes by GREAT tool.

C. GO analysis was applied to the downregulated genes in FKBP-SETD1A at day 1. The DNA damage response pathways are in blue, and the heme biosynthesis process is in red.

D. The overlap between RNA downregulated genes in MV-411;FKBP-SETD1A at 6 days of post-dTAG-13 treatment and DR genes in MOLM-13;FKBP-SETD1A cells.

E. GO analysis applied to the overlapped 49 genes in Figure S2C are shown.

F-G. Relative RNA expressions of *SETD1A* and *COX15* in SETD1A sgRNA-expressing MOLM-13, non-MLL-r leukemia cell lines (U937 and K562) and sarcoma cell lines (A673 and RH30) against empty sgRNA-expressing cells are shown. iCas9-expressing leukemia cells or Cas9-expressing sarcoma cells were cultured for 4 days post-doxycycline treatment or 6 days post-infection, respectively.

H. Relationship between *SETD1A* expression level and COX family gene expression level in the DepMap database.

I-J. RNA expressions of *SETD1A*, *HMBS*, *UROS*, *COX15* and *COX18* in AML patients from TCGA database were analyzed. Correlation between *SETD1A* and DR genes were shown in (I). Expression levels of these genes in all patients are shown in (J).

Data are represented as mean ± SD.

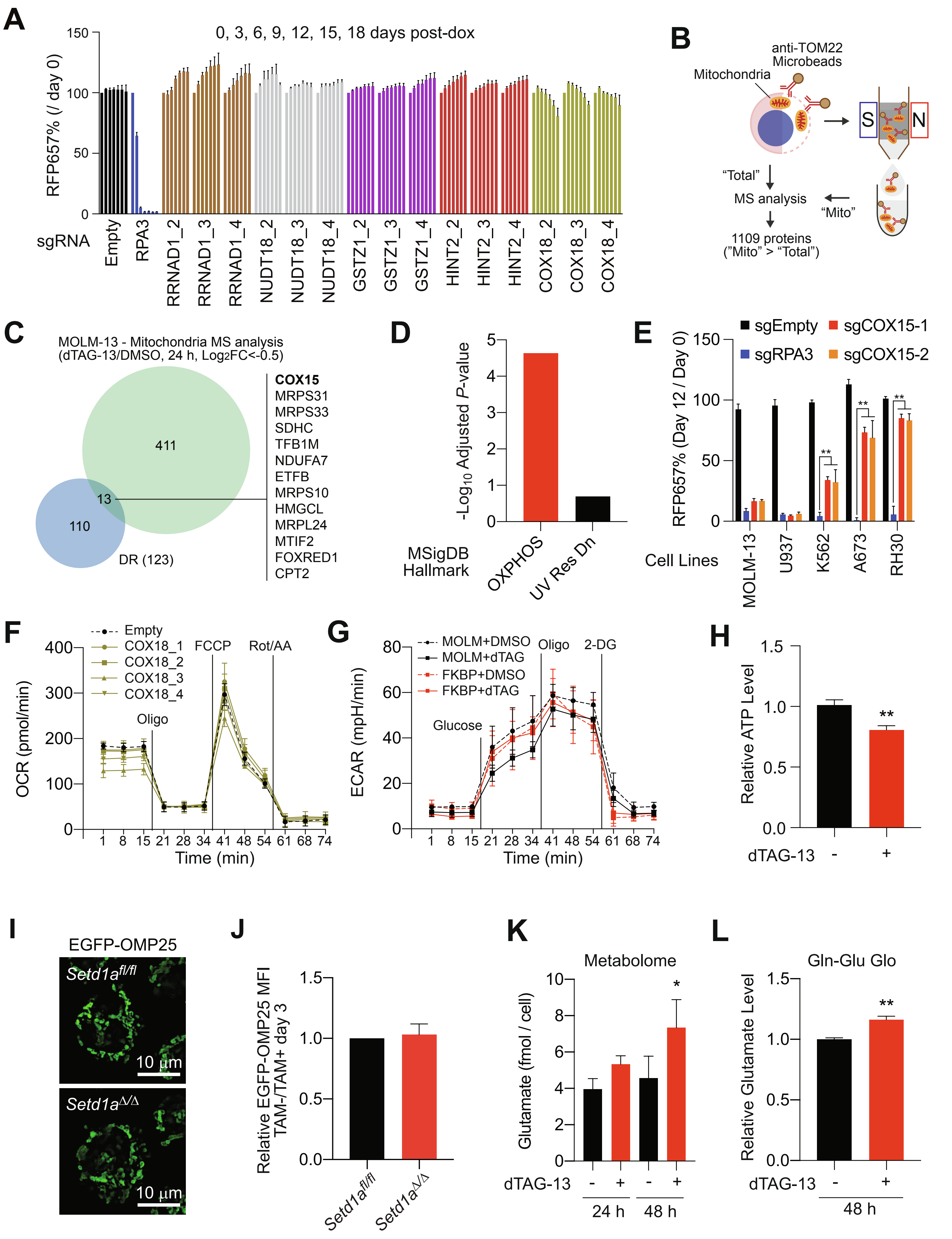

Hoshii T - Figure S3

Figure S3. SETD1A loss reduces mitochondrial respiration without affecting the glycolysis and mitochondrial mass in leukemia cells, Related to Figure 3.

A. sgRNAs for SETD1A target candidates were transfected into iCas9-expressing MOLM-13 leukemia cells. Representative data from out of 2 independent experiments with 3 biological replicates are shown.

B. Schematic illustration for the mitochondria enrichment followed by MS analysis. Mitochondria was enriched by microbeads conjugated with anti-TOM22 antibody, and "Mito" proteins were determined by the subtraction of total proteins.

C. The overlap between 123 DR genes and the downregulated 411 Mito proteins in FKBP-SETD1A cells.

D. GO analysis of 411 Mito proteins in FKBP-SETD1A cells was performed by Enrichr.

E. *COX15* sgRNAs were transfected into iCas9-expressing MOLM-13, U937 and K562 leukemia cells and Cas9-expressing A673 and RH30 sarcoma cells. Leukemia cells or sarcoma cells were cultured for 12 days post-doxycycline treatment or 15 days post-infection, respectively.

F. Oxygen consumption rates in COX18-knockout cells were evaluated by flux analyzer at 7 days post-dox treatment. Oligo (Oligomycin: ATP synthase inhibitor), FCCP (OXPHOS uncoupler) and Rot/AA (inhibitors of mitochondrial electron transport chain) were treated at indicated time points.

G. Glycolytic rates in MOLM-13 and FKBP-SETD1A cells were evaluated by flux analyzer at 72 h post-dTAG-13 treatment. Glucose, Oligo and 2-DG (2-deoxy-D-glucose) were treated at indicated time points.

H. Relative ATP level in 1×10^4 cells were evaluated by CellTiter-Glo assay.

I. The EGFP fluorescence in EGFP-OMP25-expressing *Setd1a*$^{fl/fl}$ or *Setd1a*$^{fl/fl}$*;CreER* AML cells at 3 days post-tamoxifen treatment. Scale bars = 10 µm.

J. The relative EGFP-OMP25 mean fluorescence intensity in EGFP-OMP25-expressing *Setd1a*$^{fl/fl}$ or *Setd1a*$^{fl/fl}$*;CreER* AML cells. The EGFP MFI were evaluated by flow cytometry at 3 days post-tamoxifen treatment.

K. Metabolomics was performed at 24 and 48 h post-dTAG-13 treatment in FKBP-SETD1A cells. Concentrations of glutamate per cell are shown.

L. Intracellular glutamate level was measured by Gln-Glu Glo assay at 48 h post-dTAG-13 treatment in FKBP-SETD1A cells.

Data are represented as mean ± SD.

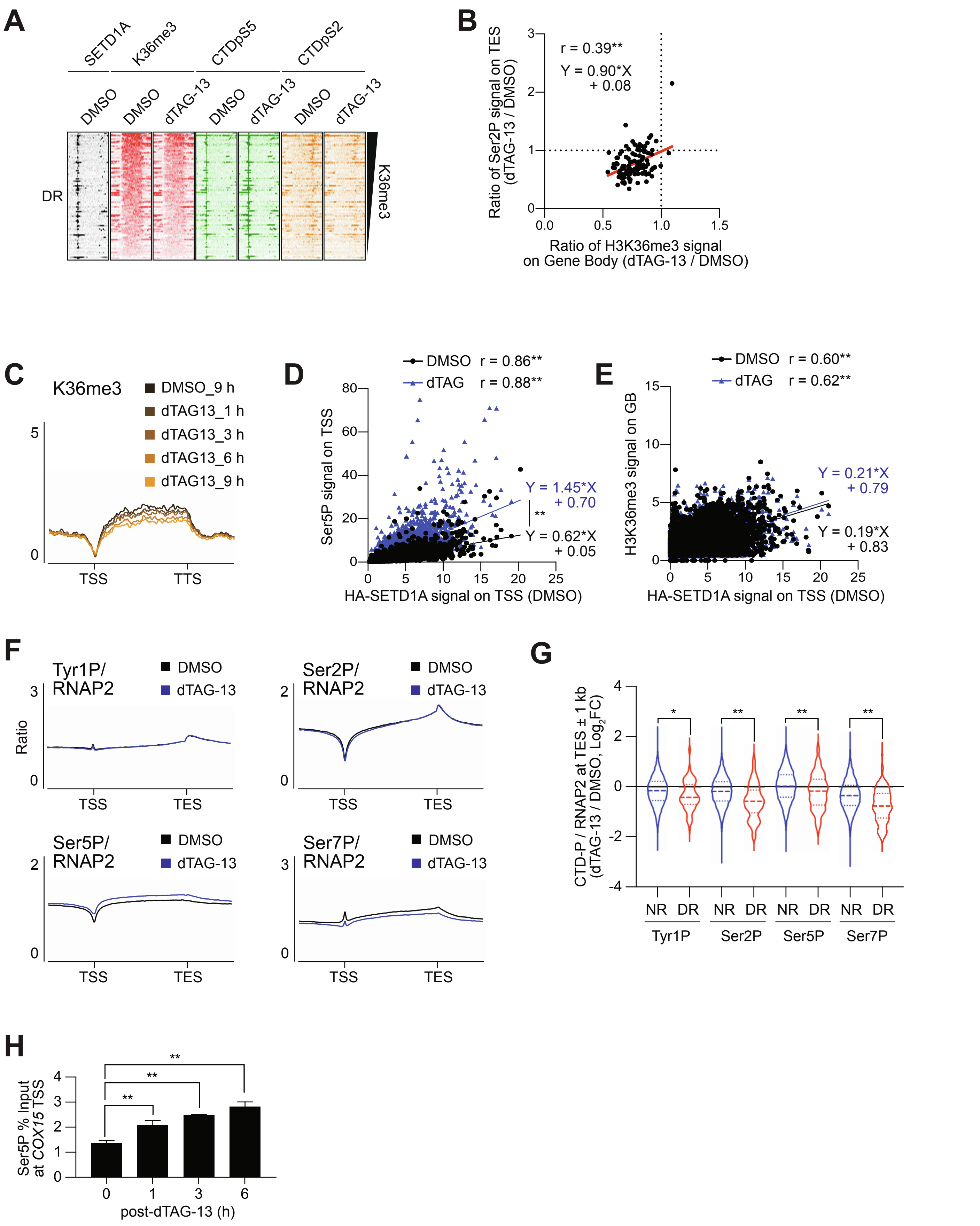

Hoshii T - Figure S4

Figure S4. DR genes showed the defective elongation phenotypes after the SETD1A degradation, Related to Figure 4.

A. Heatmaps of ChIP-seq data on SETD1A, K36me3, Ser5P and Ser2P at DR genes in DMSO or dTAG-13 treated cells are shown.

B. Correlation between relative H3K36me3 signal on gene body and the relative Ser2P signal on TTS in dTAG-13-treated FKBP-SETD1A cells are shown. Correlation coefficient (r) and *P* values of Spearman correlation as well as the simple linear regression equations are shown.

C. Average ChIP-seq signals of K36me3 at DR genes in DMSO or dTAG-13 treated FKBP-SETD1A cells. dTAG-13 treated cells were harvested at 1, 3, 6 and 9 h post-treatment.

D. Correlation of ChIP-seq signal intensity between HA-SETD1A and Ser5P at TSS on NR genes are shown. Correlation coefficient (r) and *P* values of Spearman correlation in DMSO or dTAG-13 treated cells are shown. The simple linear regression equations and the difference between two groups were also calculated.

E. Correlation of ChIP-seq signal intensity between HA-SETD1A at TSS and H3K36me3 at gene body on NR genes is shown. Correlation coefficient (r) and *P* values of Spearman correlation in DMSO or dTAG-13 treated cells are shown. The simple linear regression equations are shown. There is no significant difference between DMSO vs dTAG-13.

F. ChIP-seq signals of each RNAP2 phosphorylation normalized to total RNAP2 along NR genes and flanking regions from DMSO (black) or dTAG-13 (red) treated cells are shown.

G. Log_2FC of normalized ChIP-seq signals of each RNAP2 phosphorylation (Tyr1P, Ser2P, Ser5P and Ser7P) by total RNAP2 at TES ± 1 kb of NR or DR genes in DMSO or dTAG-13 treated samples are shown.

H. Ser5P at COX15 TSS in dTAG-13 treated cells at the indicated time points were evaluated by ChIP-qPCR.

Data are represented as mean ± SD.

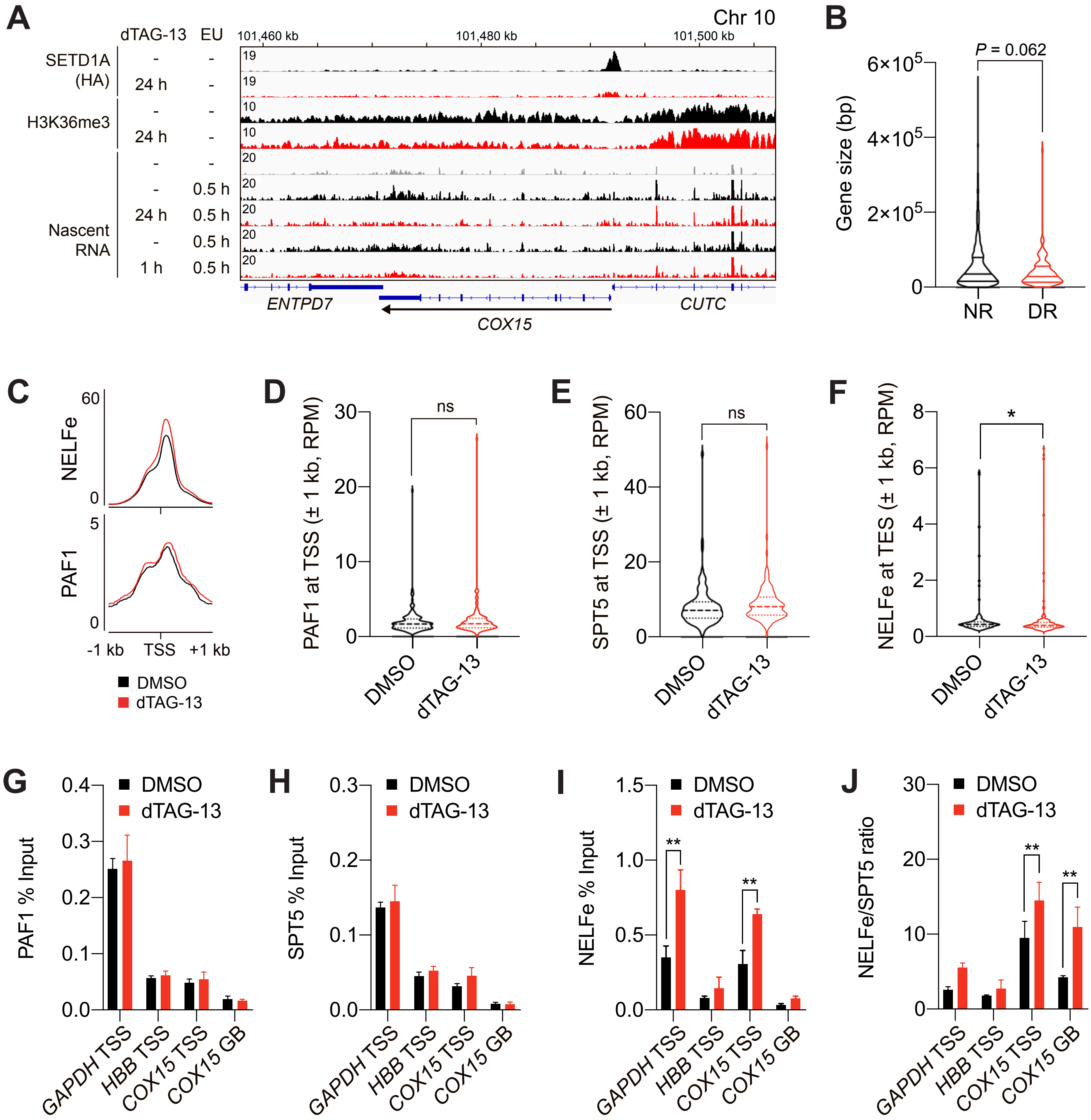

Hoshii T - Figure S5

Figure S5. SETD1A-dependent transcriptional perturbation is not associated with gene size, Related to Figure 5.

A. Browser view of HA-SETD1A and K36me3 ChIP-seq data and the nascent RNA-seq data, at *COX15* locus. Each samples were treated with DMSO (-) or 500 nM dTAG-13, followed by 500 nM EU, for indicated time.

B. Average gene size of NR and DR genes.

C. Average ChIP-seq signals of NELFe and PAF1 at TSS ± 1kb of DR genes from DMSO (black) or dTAG-13 (red) treated FKBP-SETD1A cells are shown.

D-F. The intensities (RPM) of PAF1 (D) and SPT5 (E) at TSS ± 1kb, and NELFe (F) at TES ± 1kb of DR genes in DMSO or dTAG-13 treated FKBP-SETD1A cells are shown.

G-J. Localization of PAF1 (G), SPT5 (H) and NELFe (I) at the indicated loci in DMSO or dTAG-13 treated FKBP-SETD1A cells are evaluated by ChIP-qPCR. NELFe/SPT5 ratio (J) were calculated from % input of individual factors.

Data are represented as mean ± SD.

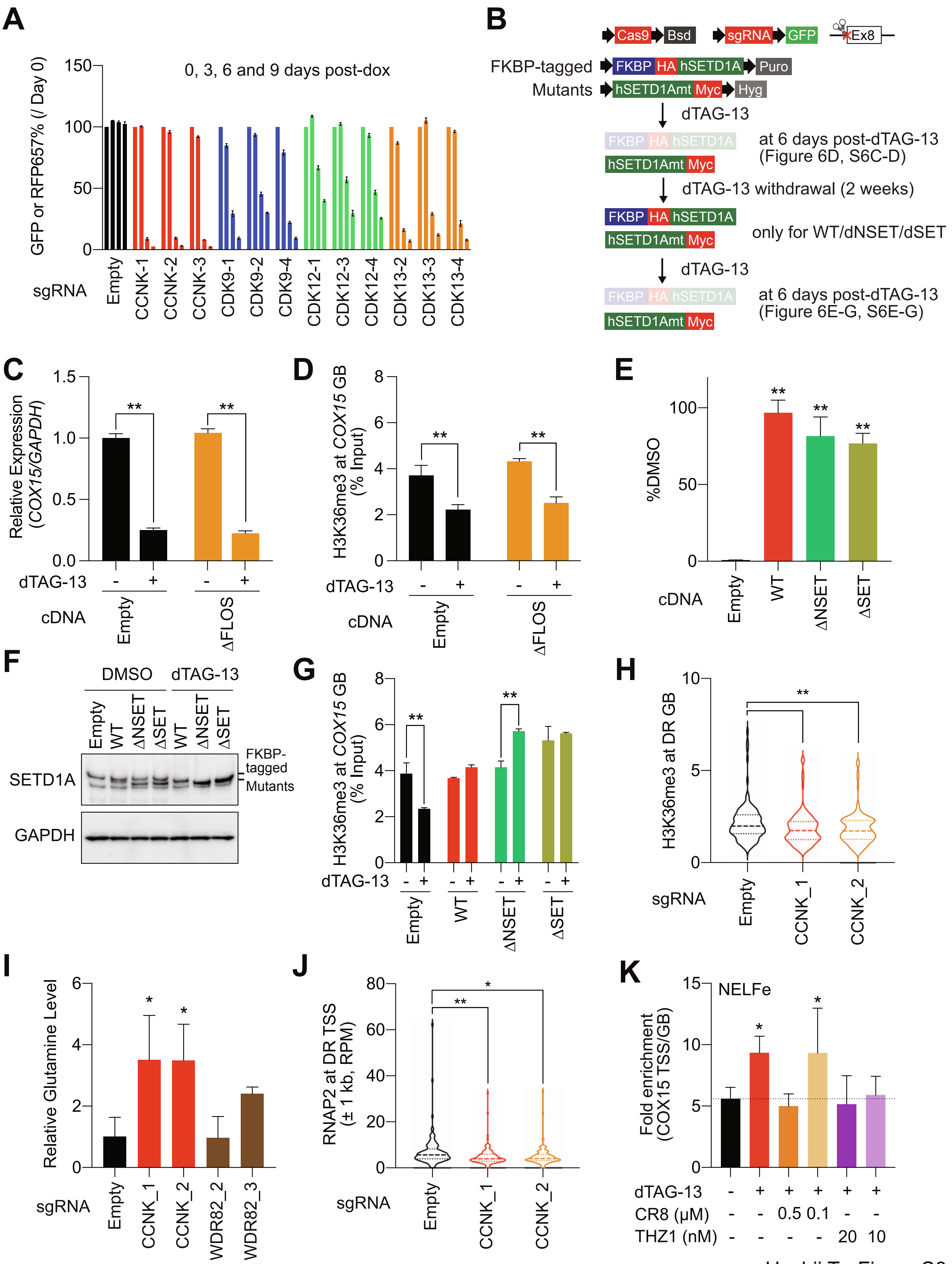

Hoshii T - Figure S6

Figure S6. Cyclin K is required for heme biosynthesis pathway, mitochondrial respiration as well as the RNAP2 pausing at DR genes, Related to Figure 6.

A. sgRNAs for CCNK, CDK9, CDK12 and CDK13 were transfected into iCas9-expressing MOLM-13 leukemia cells. Representative data from out of 2 independent experiments with 3 biological replicates are shown.
B. Schematic illustration of the human SETD1A rescue experiments in human FKBP-SETD1A cells.
C. *COX15* transcriptional level in SETD1A ΔFLOS mutant expressing FKBP-SETD1A cells was monitored by qRT-PCR at 24 h post-dTAG-13 treatment.
D. H3K36me3 level at *COX15* locus in SETD1A ΔFLOS mutant-expressing FKBP-SETD1A cells was monitored by ChIP-qPCR
E. dTAG-13 treated and withdrawn human SETD1A deletion mutant-expressing FKBP-SETD1A cells were treated again with dTAG-13 and cell counts were performed at 6 days post-treatment.
F. Western blot analysis was performed to check the expression of FKBP-SETD1A and SETD1A mutants at 6 days post-dTAG-13 treatment.
G. H3K36me3 level at *COX15* locus in SETD1A deletion mutant-expressing FKBP-SETD1A cells was monitored by ChIP-qPCR
H. H3K36me3 intensities (RPM) at DR gene bodies in cyclin K-knockout cells were evaluated from ChIP-seq data, which are shown in Figure 5I.
I. Relative Gln level in cyclin K- or WDR82-knockout cells were analyzed at 4 days post-dox treatment.

J. Total RNAP2 level (RPM) at TSS ± 1kb of DR genes in cyclin K-knockout cells was evaluated by ChIP-seq.

K. ChIP was performed with NELFe (L) antibodies in dTAG-13/CR8/THZ1 treated FKBP-SETD1A cells at 24 h post-treatment, and immunoprecipitated DNA was quantified by qPCR with specific primers for *COX15* locus.

Data are represented as mean ± SD.

Table S1. A list of sgRNA used in this study, Related to the STAR Methods section

sgRNAs		
Name of sgRNA	sgRNA sequence	Notes
SETD1A-Ex8	AAGAGCATGGAGAAGCCTGC	Target intron7-exon8 junction of SETD1A gene
RPA3	GATGAATTGAGCTAGCATGC	positive control
RRNAD1_1	GGTCTACCCACCTAACCACG	
RRNAD1_2	CAGCCACCCACTGACTCAGT	
RRNAD1_3	GGCAACACTCAGATCCCCAC	
RRNAD1_4	GATGGTGAAGAGCATCGAAG	
HINT2_1	TCACGGAACACAAGACACTG	
HINT2_2	TCTTCAGCCTGGCTAATCCG	
HINT2_3	GGAATGACCAGGAAGTGCAC	
HINT2_4	CCTCATAGAGAATGTCAGCT	
COX15_1	GAGTACTCACACCGAATGTG	
COX15_2	GCTGGCACAATAAAGAACCA	
COX15_3	AATGCCAATCTACCATCGAG	
COX15_4	GTCATATCATGATTCAAGCT	
NUDT18_1	AAGTCTTGAGAATTCCACCT	
NUDT18_2	AGGATGTCATGGGCTCGCAG	
NUDT18_3	GGGTGTTAGTGGGCACAGTG	
NUDT18_4	CGCTGGCAGACCAGATCACA	
GSTZ1_1	GAAAGGCATCGACTACGAGA	
GSTZ1_2	GTCAGAAATCATACGCACGC	
GSTZ1_3	CTGTCTGTCCTGAAGCAAGT	
GSTZ1_4	CTCCTCTAGATACTCAATGA	

Table S1. A list of sgRNA used in this study (continued)

COX18_1	CAGGAACAGTTAGCTACTGG	
COX18_2	TTTGGATTCAGCTTCCAATG	
COX18_3	ACATGCGAACGGCTGGTACG	
COX18_4	TTCGTGCAAATCAGTTGGGG	
HMBS_1	GGCTCGCACTTCCACGCCCA	
HMBS_2	TGCTTCGCTGCATCGCTGAA	
HMBS_3	CGTGGGTACCCGCAAGAGCC	
HMBS_4	GGGCGTGGAAGTGCGAGCCA	
UROS_1	GTGGCCAGGATCCGTATATC	
UROS_2	CCAGCGCGCGAGCCGTAGTG	
UROS_3	CGATAAAACAGGGATCAAAG	
UROS_4	TCCAACCACATACACTGACT	
CCNK_1	GTAGCAGTGATGTATCTCGC	
CCNK_2	GGACCTCCAAACCCATGTAT	
CCNK_3	GTAGCAGTGATGTATCTCGC	
CCNK_4	GGGGCTACACTATGATACCC	
CDK12_1	CCGAAGTGTTGTTAACATGA	
CDK12_2	GTTTCATGAACGACTTGATA	
CDK12_3	AACACTTAATATCCCGATGC	
CDK12_4	CGACCTCCAGAACTACTGCT	
CDK13_1	CCATGATCTGATGGGACTAC	
CDK13_2	ATTTATGAGACAGCTCATGG	
CDK13_3	CGTCCACCTGAACTGCTACT	
CDK13_4	TTTTAGTGAAGAGTTCGCCA	

Table S1. A list of sgRNA used in this study (continued)

CDK9_1	AAGCTCGCCAAGATCGGCCA	
CDK9_2	GCACCGCAAGACCGGCCAGA	
CDK9_3	CCACCTTCTGGCCGGTCTTG	
CDK9_4	AAGGATCTTGATCTCCCGCA	
WDR82_1	TTTCAGCCCCAACGGCGAGA	
WDR82_2	GTACAGTAAGAAATATGGTG	
WDR82_3	AGGGCCTCATGCATCTGCAG	
WDR82_4	TACAGCTCTAACAAAATAGA	
SSU72_1	GCGTCCGATCCTTTGGAACA	
SSU72_2	AGAGAGAGTGTATGACCAGG	
SSU72_3	ATTGTACATCTGGTCATATG	
SSU72_4	GGGTGGAGCCCAACTACCTG	

Table S2. A list of oligonucleotide primers used in this study, Related to the STAR Methods section

Purpose	Name	Sequence
Human *COX15* mRNA expression	hCOX15-RT-fwd	GCTTGTGGACCTCACTGTCA
Human *COX15* mRNA expression	hCOX15-RT-rev	AGACCTGCTGTTCCATGAGC
Human *HMBS* mRNA expression	hHMBS-RT-fwd	GAGACTCTGCTTCGCTGCAT
Human *HMBS* mRNA expression	hHMBS-RT-rev	CAGTCAGGTACAGTTGCCCA
Human *UROS* mRNA expression	hUROS-RT-fwd	AGTCCTCAGCACTGCCTCTT
Human *UROS* mRNA expression	hUROS-RT-rev	CTGGGTGTGCAACTGTCTGA
ChIP-qPCR for *HBB* TSS	hHBB-TSS-fwd	CAGGGTGAGGTCTAAGTGATGA
ChIP-qPCR for *HBB* TSS	hHBB-TSS-rev	TTGAAGTCCAACTCCTAAGCCA
ChIP-qPCR for *GAPDH* TSS	hGAPDH-TSS-fwd	CCACTAGGCGCTCACTGTTCTC
ChIP-qPCR for *GAPDH* TSS	hGAPDH-TSS-rev	GAACTCACCCGTTGACTCCGAC
ChIP-qPCR for *COX15* TSS	hCOX15-TSS-fwd	CTCCGCGCTCGACAATTAAC
ChIP-qPCR for *COX15* TSS	hCOX15-TSS-rev	CACACGAGGATGCCAGTACC
ChIP-qPCR for *COX15* gene body	hCOX15-GB-fwd	GGTGAGTTAGAAACCTGGCACT
ChIP-qPCR for *COX15* gene body	hCOX15-GB-rev	AGGAAGTAGAGCACTGTAATGGC
DRB-release assay for *COX15* locus	hCOX15-intron1-fwd	GGGATAAAGATAATGTAGAGCCCCA
DRB-release assay for *COX15* locus	hCOX15-intron1-rev	GAACCTATGCGTTGTGAGTGC
